# Spatio-temporal patterns and prediction of colorectal cancer mortality in Chinese cancer registration areas: A nationwide study based on multiple models

**DOI:** 10.1371/journal.pone.0352110

**Published:** 2026-07-02

**Authors:** Yu Zhao, Xiaoshuang He, Jianjiang Shao, Lei Zhu, Zhiqiang Chu, Yang Man, Jiageng He

**Affiliations:** 1 Department of Gastrointestinal Surgery, The First Affiliated Hospital of Shihezi University, Shihezi, Xinjiang, China; 2 Department of Pulmonary and Critical Care Medicine, The First Affiliated Hospital of Shihezi University, Shihezi, Xinjiang, China; 3 School of Public Health, Shihezi University, Shihezi, Xinjiang, China; Faculdade Ciencias Medicas de Minas Gerais, BRAZIL

## Abstract

**Objective:**

To describe the spatiotemporal distribution characteristics of colorectal cancer (CRC) mortality in Chinese cancer registration areas and to predict its future trends, thereby providing an epidemiological basis for CRC prevention and control in China.

**Method:**

This study used cancer registry data from 2005 to 2018, collected from the China Cancer Registry Annual Report (2008–2021), to comprehensively describe CRC mortality patterns. Joinpoint regression models were employed to calculate the annual percentage change (APC) and average annual percentage change (AAPC) to analyze temporal trends. The age–period–cohort model was used to disentangle the effects of age, period, and cohort. A Bayesian age–period–cohort (BAPC) model was constructed to predict future trends for 2019–2035. Global and local spatial autocorrelation methods were applied to describe the spatial distribution of CRC mortality and to identify high-risk areas.

**Results:**

From 2005 to 2018, in Chinese cancer registration areas, the mortality of CRC was 14.45/100,000, and the age-standardized mortality rate (ASMR) was 11.13/100,000 [95% confidence interval (CI): 11.03, 11.27]. From 2005 to 2007, the mortality rate of CRC showed an upward trend, with an APC of 3.67% (95% CI: −0.77%, 8.33%); from 2007 to 2013, there was a significant downward trend with an APC of −2.45% (95%CI: −3.14%, −1.47%); from 2013 to 2018, there was an upward trend with an APC of 0.13% (95% CI: −0.88%, 1.17%). From 2007 to 2013, the CRC mortality rates of different genders showed a significant downward trend. In urban areas from 2009 to 2018, the CRC mortality rate showed a significant downward trend with an APC of −1.21% (95% CI: −1.71%, −0.71%), but in rural areas, it showed a significant upward trend with an AAPC of 0.59% (95% CI: 0.24%, 1.56%). The age effects indicated that the risk of CRC death increased with age. From 2019 to 2035, the ASMR of CRC in Chinese cancer registration areas will generally show a slow upward trend. Spatial autocorrelation analysis showed that the mortality rate of CRC in Chinese cancer registration areas exhibited spatial clustering characteristics, with hotspots concentrated in the eastern coastal and northeastern provinces.

**Conclusions:**

The mortality rate of CRC in Chinese cancer registration areas showed a trend of first increasing, then decreasing, and then increasing from 2005 to 2018. It was predicted that it will continue to rise from 2019 and still face a series of challenges such as urban-rural, gender, and regional inequality. In the future, more targeted prevention and control strategies should be adopted, combined with dietary and lifestyle interventions, to further reduce the burden of CRC.

## Introduction

In recent years, with the intensification of population aging and rapid economic development, cancer has become a major global public health challenge, and cancer- related deaths have emerged as the leading cause of mortality [1]. According to the latest report from the International Agency for Research on Cancer (IARC), in 2022, 18.7 million new cancer cases and 9.7 million cancer deaths were reported in 183 countries worldwide, representing increases of 31.4% and 20.3%, respectively, compared with 2015 [2]. By 2050, the numbers of global cancer cases and deaths were projected to rise to 33 million and 18 million, respectively—an increase of 76.5% and 85.6% compared with 2022 [3]. Cancer not only severely threatens patients health and survival but also imposes a heavy burden on families and society.

Colorectal cancer (CRC) is one of the major cancers that threatens human health. According to the Global Burden of Disease (GBD) report, in 2022, there were approximately 1.87 million new CRC cases worldwide, ranking third among cancers, and about 882,000 deaths, ranking second in cancer-related mortality [4]. The incidence of CRC is closely related to socioeconomic status—countries with a higher social development index (SDI) usually show higher incidence. However, with the advancement of CRC diagnosis and treatment, incidence and mortality have stabilized or declined in developed countries over recent decades, while rapidly increasing in most low- and middle-income countries [5]. The 2022 China Cancer Statistics report revealed that the incidence and mortality of CRC in China ranked second and fourth, respectively, among all cancers, and by 2030, CRC mortality was expected to rise to second place [6]. These data highlight that the mortality of CRC remain at high levels in China, making CRC a significant public health burden that cannot be ignored.

A comprehensive understanding of the epidemiological characteristics of CRC is a prerequisite for effective prevention and treatment. Therefore, this study collected CRC mortality data from 2005 to 2018 reported in the China Cancer Registry Annual Reports (2008–2021), and described the mortality patterns in Chinese cancer registration areas across 14 years and 33 different regions for both genders from a national perspective. We used the Joinpoint regression model to examine temporal trends, the age–period–cohort model to assess the effects of age, period, and birth cohort on CRC mortality, and the Bayesian age–period–cohort (BAPC) model to predict CRC mortality trends from 2019 to 2035. Additionally, spatial autocorrelation analysis was conducted on CRC mortality across 33 provinces. Together, these analyses comprehensively characterized the epidemiological features of CRC and provided scientific evidence for the development of targeted prevention and control strategies in China.

## Materials and methods

### Data sources

CRC mortality data from 2005 to 2018 were obtained from the China Cancer Registry Annual Reports (2008–2021) [7–20]. Standard population data were derived from the 2010 Sixth National Population Census published by the National Bureau of Statistics of China [21]. CRC cases were diagnosed based on the International Classification of Diseases, 10th Revision (ICD-10/C18–C21) [22].

### Data quality

To ensure the authenticity, stability, and comparability of the data, data collection, verification, and analysis were conducted in accordance with the Chinese Cancer Registry Guidelines and the standards of the IARC, ensuring consistency in registration procedures and coding practices across regions and study years. All registries underwent standardized evaluations of comparability, completeness, and validity.

The inclusion and exclusion of data strictly adhered to the quality control standards for Chinese cancer registration work. Data included in the report must meet the following core quality requirements: the morphological verification percentage (MV%) should range between 55% and 95%; the percentage of death certificate only (DCO%) should be less than 20%; the mortality-to-incidence ratio (M/I) should fall within the range of 0.55 to 0.85.

From 2008 to 2021, the coverage of cancer registries reported in the China Cancer Registry Annual Report expanded steadily. It increased from 45 cancer registries in 2005, covering a population of 69,369,668 (accounting for 5.31% of the national population at the end of 2005), to 947 registries by 2018, covering 634,376,540 individuals (representing 45.14% of the national population at the end of 2018) (Table 1). From 2005 to 2018, the MV%, DCO%, and the M/I were 67.83%, 2.11%, and 0.62, respectively, all of which met national quality benchmarks (Table 2). These indicators collectively demonstrate that the registry data are robust and reliable, providing a sound foundation for epidemiological analysis.

**Table 1:** Number of cancer registries and population coverage in China, 2005–2018.

Year	Number of cancer registries	Covered population	Population coverage (%)
2005	45	69,369,668	5.31
2006	49	76,209,748	5.80
2007	48	70,782,375	5.36
2008	56	82,433,497	6.21
2009	104	109,476,347	8.20
2010	219	207,229,403	15.42
2011	234	221,390,275	16.43
2012	261	239,887,749	17.64
2013	347	287,284,044	21.11
2014	449	345,711,646	25.27
2015	501	387,872,825	28.22
2016	682	476,692,113	34.47
2017	821	563,934,185	40.57
2018	947	634,376,540	45.14

**Table 2:** Quality indicators of cancer registry data in China, 2005–2018.

Year	National			Urban			Rural		
	MV(%)	DCO(%)	M/I	MV(%)	DCO(%)	M/I	MV(%)	DCO(%)	M/I
2005	66.00	1.76	0.65	69.02	2.08	0.65	56.09	0.71	0.28
2006	65.59	1.66	0.64	67.71	1.85	0.61	57.11	0.90	0.76
2007	65.83	1.95	0.64	67.71	2.28	0.61	59.57	0.83	0.75
2008	69.33	2.23	0.62	70.53	2.49	0.59	64.22	1.12	0.73
2009	67.23	3.14	0.63	68.96	3.03	0.60	62.91	3.43	0.71
2010	67.11	2.99	0.61	71.51	2.49	0.59	60.65	3.72	0.64
2011	70.14	2.44	0.63	72.92	2.17	0.61	65.34	2.90	0.67
2012	69.13	2.38	0.62	70.63	2.63	0.59	67.31	2.09	0.65
2013	68.04	1.74	0.62	70.76	1.76	0.59	64.97	1.72	0.65
2014	68.01	2.91	0.61	69.75	2.79	0.58	65.96	1.49	0.64
2015	39.31	2.08	0.61	64.45	3.25	0.53	62.32	2.92	0.63
2016	68.31	1.40	0.61	70.03	1.58	0.59	66.29	1.18	0.63
2017	67.77	1.53	0.59	69.97	1.62	0.56	65.47	1.44	0.62
2018	69.94	1.34	0.58	71.50	1.58	0.55	68.48	1.11	0.61
AVG	65.84	2.11	0.62	69.68	2.26	0.59	63.34	1.83	0.64

MV%, morphological verification percentage; DCO%, percentage of death certificate only; M/I, mortality-to-incidence ratio.

### Classification standards

The urban-rural classification in this study was based on the administrative division codes of the People’s Republic of China (GB/T2260-2007), with cities at or above the prefecture level classified as urban areas and counties and county-level cities classified as rural areas.

### Statistical indicators

Mortality rate, also known as crude mortality rate, refers to the number of cancer deaths registered per 100,000 population in a given year, and reflects the mortality level of the population. Since the crude mortality rate is significantly affected by the age composition of the population, when comparing and analyzing mortality rates across different regions or the mortality levels of the same population group over different periods, it is necessary to calculate the age-standardized mortality rate (ASMR) to eliminate the impact of population age structure on mortality. The ASMR refers to the mortality rate calculated according to the age structure of a standard population. In this study, mortality data across the country, different genders, and different regions were divided into 18 age groups with an interval of 5 years: 0–4 years, 5–9 years, 10–14 years...80–84 years, and 85 years and above. The standard population data came from the sixth national population census data released by the National Bureau of Statistics of China in 2010 [21]. In this study, ASMR were used in the Joinpoint regression and BAPC models to ensure comparability over time and across populations. For provincial-level spatial analysis, crude mortality rates were used due to the lack of age-specific data in China Cancer Registry Annual Reports.


$$\begin{array}{c} \text{Mortality rate per 100},\text{000}=\frac{\text{count}}{\text{population}}\times \text{100},\text{000} \end{array}$$

$$\begin{array}{c} \text{ASMR}=\frac{\sum \text{standard population in corresponding age group }\times \text{ age}-\text{specific mortality rate}}{\sum \text{standard population}}\times \text{100},\text{000} \end{array}$$

The 95% confidence interval (CI) for the ASMR was estimated using the Fay-Feuer method, which is robust for directly standardized rates across varying population sizes and incidence levels [23].


$$\begin{array}{c} \text{ASMR 95}\%\text{C}{\text{I}}_{\text{low}}=\left(\frac{\text{v}}{\text{2}\times \text{ASMR}}\right)\times \left(\text{ChiInv}\left(\frac{\text{0}.\text{05}}{\text{2}},\frac{(\text{2}\times {\text{ASMR}}^{\text{2}})}{\text{v}}\right)\right)\times \text{100},\text{000} \end{array}$$

$$\begin{array}{c} \text{ASMR 95}\%\text{C}{\text{I}}_{\text{high}}=\left(\frac{\text{v}+\overset{\text{2}}{\underset{\text{m}}{\text{w}}}}{\text{2}\times (\text{ASMR}+{\text{w}}_{\text{m}})}\right)\times \left(\text{ChiInv}\left(\text{1}-\frac{\text{0}.\text{05}}{\text{2}},\frac{\text{2}(\text{ASMR}+{\text{w}}_{\text{m}}{)}^{\text{2}}}{\text{v}+\overset{\text{2}}{\underset{\text{m}}{\text{w}}}}\right)\right)\times \text{100},\text{000} \end{array}$$

Where, ${\text{w}}_{\text{m}}$ is the weight correction factor, v is the estimated variance of the ASMR, ChiInv (p, n) is the inverse of the chi-squared distribution function evaluated at p and with n degrees of freedom.

### Data analysis

#### Joinpoint regression model.

Joinpoint regression was applied to analyze temporal trends in CRC mortality. This method fits segmented regression models to time-series data and identifies points where statistically significant changes in trends occur. Given that cancer mortality data typically follow a Poisson or exponential distribution, a log-linear Joinpoint model was employed in this study [24,25]. The model can be expressed as:


$$\begin{array}{c} \text{ln}\left(R_{y}\right)=\beta_{\text{0}}+\beta_{\text{1}}y+\sum_{k=\text{1}}^{K}\delta_{k}{\left(y-\tau_{k}\right)}^{+} \end{array}$$

Where, $R_{y}$ denotes the CRC mortality (per 100,000 population) in year y, $\beta_{\text{0}}$ is the intercept, $\beta_{\text{1}}$ is the baseline slope, $\tau_{\text{k}}$ represents the location of the k-th joinpoint, and ($y-\tau_{k}$)+=max(0, $y-\tau_{k}$). The number of joinpoints (K) was set to four in this study.

Model selection was performed using the Monte Carlo permutation test, which identifies the optimal number of joinpoints by comparing goodness-of-fit across models.

The period 2005–2018 was divided into multiple segments. The annual percentage change (APC) was used to evaluate within-segment trends, while the average annual percentage change (AAPC) described the overall trend. A 95% CI was used to assess statistical significance. When the number of joinpoints equals zero, APC = AAPC. APC > 0 or AAPC>0 indicates an increasing trend, while APC < 0 or AAPC<0 indicates a decreasing trend.

APC calculation formula:


$$\begin{array}{c} \text{APC}=\left({\text{e}}^{\beta_{\text{1}}}-\text{1}\right)\times \text{1}\text{0}\text{0} \end{array}$$

AAPC calculation formula:


$$\begin{array}{c} \text{AAPC}=\left[\text{exp}\left(\sum \omega_{\text{i}}\beta_{\text{i}}/\sum \omega_{\text{i}}\right)-\text{1}\right]\times \text{1}\text{0}\text{0} \end{array}$$

In the formula, $\beta_{\text{1}}$ is the regression coefficient, $\beta_{\text{i}}$ is the regression coefficient corresponding to each interval and $\omega_{\text{i}}$ is the span of each interval (i.e., the number of years included in the interval).

#### Age–period–cohort model.

The age–period–cohort model is based on the Poisson distribution and is used to identify the independent effects of age, period, and cohort on observed variables [26]. The model is expressed as:


$$\begin{array}{c} log\left(\lambda_{apc}\right)=\alpha_{a}+\beta_{p}+\gamma_{c}+\epsilon \end{array}$$

In the formula, α、β and γ represent the effects of age, period, and cohort, while ε represents the residual.

According to the requirements of the age-period-cohort model, the data needs to be organized into the following format

#### Age group.

This study included data on CRC deaths in various age groups from 2005 to 2018. The CRC death cases in China were divided into 18 age groups, ranging from 0–4 years, 5–9 years...up to 85 years and above, based on the age group of every 5 years.

#### Period group.

In order to comply with the norms of the age-period-cohort model, this study divided the time span from 2005 to 2018 into periods every 5 years, resulting in a total of 3 periods: 2005–2009, 2010–2014, and 2015–2018.

#### Cohort group.

In the age-period-cohort model, the birth cohort was obtained by subtracting age from period. Therefore, this study defined 20 birth cohorts from 1920–1924–2015–2019.

The results of age, period, and cohort effect are usually measured by relative risk (RR) to assess changes in the risk of death in different age groups, periods, or birth cohorts relative to the reference group, where RR > 1 indicates increased risk and RR < 1 indicates decreased risk.

#### Bayesian age–period–cohort model.

To address the identifiability problem in the traditional age–period–cohort model— arising from the exact linear dependency among age, period, and cohort—this study employed a BAPC model to perform smoothed estimation and predict the long-term trend of CRC mortality. The BAPC model incorporates Bayesian random effects and smoothing priors within the age–period–cohort framework, providing stable parameter estimates while preserving the interpretability of the three temporal factors [27,28]. Unlike the intrinsic estimator (IE) approach commonly used in traditional age–period–cohort analysis, the BAPC model addresses the identifiability problem through Bayesian smoothing priors (e.g., second-order random walk priors), which impose structural constraints on adjacent parameters. This approach allows for stable and interpretable estimation of age, period, and cohort effects without requiring explicit identification restrictions.

#### Model specification.

Let ${\text{Y}}_{\text{ap}}\;$ denote the number of CRC cases in age group a and period p, and ${\text{E}}_{\text{ap}}$ denote the corresponding population at risk. We assumed:


$$\begin{array}{c} {\text{Y}}_{\text{ap}}\sim \text{Poisson}\left({\text{E}}_{\text{ap}}\lambda_{\text{ap}}\right) \end{array}$$

Where, $\lambda_{\text{ap}}$ represents the mortality rate for the age–period unit.

The log-linear form of the model is expressed as:


$$\begin{array}{c} \text{log}\left(\lambda_{\text{ap}}\right)=\mu +\alpha_{\text{a}}+\beta_{\text{p}}+\gamma_{\text{c}} \end{array}$$

Where, μ is the intercept, $\alpha_{\text{a}}$, $\beta_{\text{p}}$ and $\gamma_{\text{c}}$ denote the age, period, and cohort effects, respectively, and c = p-a.

#### Prior distributions.

To ensure model smoothness and identifiability, the age, period, and cohort effects were assigned second-order random walk (RW2) priors:


$$\begin{array}{c} \text{p}\left(\alpha |{\text{k}}_{\alpha }\right)\propto \overset{\left(\text{I}-\text{2}\right)/\text{2}}{\underset{\alpha }{\text{k}}}\text{exp}\left(-\frac{{\text{k}}_{\alpha }}{\text{2}}\sum_{\text{i}=\text{3}}^{\text{I}}{\left(\alpha_{\text{i}}-\text{2}\alpha_{\text{i}-\text{1}}+\alpha_{\text{i}-\text{2}}\right)}^{\text{2}}\right) \end{array}$$

$$\begin{array}{c} \text{p}\left(\beta |{\text{k}}_{\beta }\right)\propto \overset{\left(\text{J}-\text{2}\right)/\text{2}}{\underset{\beta }{\text{k}}}\text{exp}\left(-\frac{{\text{k}}_{\beta }}{\text{2}}\sum_{\text{j}=\text{3}}^{\text{J}}{\left(\beta_{\text{j}}-\text{2}\beta_{\text{j}-\text{1}}+\beta_{\text{j}-\text{2}}\right)}^{\text{2}}\right) \end{array}$$

$$\begin{array}{c} \text{p}\left(\gamma |{\text{k}}_{\gamma }\right)\propto \overset{\left(\text{K}-\text{2}\right)/\text{2}}{\underset{\gamma }{\text{k}}}\text{exp}\left(-\frac{{\text{k}}_{\gamma }}{\text{2}}\sum_{\text{k}=\text{3}}^{\text{K}}{\left(\gamma_{\text{k}}-\text{2}\gamma_{\text{k}-\text{1}}+\gamma_{\text{k}-\text{2}}\right)}^{\text{2}}\right) \end{array}$$

Here, ${\text{k}}_{\alpha }$, ${\text{k}}_{\beta }$, and ${\text{k}}_{\gamma }$ are the precision parameters controlling the smoothness of age, period, and cohort effects, respectively. To reflect weakly informative prior assumptions, their hyperpriors were defined as:


$$\begin{array}{c} {\text{k}}_{\alpha },{\text{k}}_{\beta },{\text{k}}_{\gamma }\sim \text{Gamma}\left(\text{1},\text{0}.\text{0005}\right) \end{array}$$

This prior structure is widely applied in BAPC modeling and effectively balances over-smoothing and overfitting.

#### Model implementation and posterior inference.

The model was implemented in R 4.4.0 using the BAPC and INLA (Integrated Nested Laplace Approximation) packages [29,30]. The INLA algorithm was applied to approximate posterior distributions and to obtain the posterior means and 95%CI for the parameters.

In this study, the BAPC model was used to predict the ASMR of CRC in China from 2019 to 2035, stratified by sex and region. The ages were categorized into consecutive 5-year groups, which was consistent with the standard practices in age–period–cohort analysis. Model fit was evaluated using mean squared error (MSE), mean absolute error (MAE), and mean absolute percentage error (MAPE).

In addition, to assess the robustness and predictive performance of the BAPC model, a sensitivity analysis was conducted using historical data. Specifically, ASMR of CRC in China from the Global Burden Of Disease (GBD) study during 2005–2018 were used for validation.

#### Spatial autocorrelation analysis.

Spatial autocorrelation is a measure used to assess whether there is a dependency between the values of a variable within a study area and across different spatial units. This includes global and local spatial autocorrelation analyses, with common indices such as Moran’s *I*, Geary’s C, and the G statistic. This study used the Moran’s *I* statistic for both global and local spatial autocorrelation analysis [31].

The global Moran’s *I* index reflects the spatial distribution state of CRC mortality across the entire country. The value range of *I* is [−1, 1]. When its value is > 0 and closer to 1, it indicates strong positive spatial autocorrelation, meaning CRC mortality shows obvious clustered distribution; conversely, when *I* is < 0 and closer to −1, it indicates stronger negative spatial autocorrelation, meaning CRC mortality shows a discrete, mutually exclusive distribution.

The local Moran’s *I* statistic is used to test the correlation between a specific unit and its surrounding units. When the local Moran’s *I* index is closer to 0, it indicates that the mortality distribution around that spatial unit tends to be random; when the local Moran’s *I* index is positive, it indicates that the area is a high-value cluster (High-High), i.e., a “hotspot”; when the local Moran’s *I* index is negative, it indicates that the area is a low-value cluster (Low-Low), i.e., a “cold spot.” Furthermore, local spatial autocorrelation analysis can reveal anomalous areas of disease occurrence through data visualization, such as High-Low and Low-High value areas.

### Statistical analysis

This study used Joinpoint Regression Program version 5.0.2 software for Joinpoint regression analysis to describe the trend of CRC mortality in Chinese cancer registration areas from 2005 to 2018. An online web analysis tool was used to construct the age-period-cohort model. The BAPC and INLA packages in R 4.4.0 software version 4.3.2 were used to predict the trend of CRC mortality in Chinese cancer registration areas from 2019 to 2035. ArcGIS 10.8 software was used to analyze the spatial distribution characteristics of CRC mortality in Chinese cancer registration areas from 2010 to 2016. The significance level was set at *P* < 0.05.

### Ethics approval and consent to participate

This study utilized aggregated public data extracted from the China Cancer Registry Annual Report, which could be accessed on the website of the National Cancer Center of China [32]. These reports do not contain personal-level or identifiable information. Therefore, this study did not require ethical approval or additional authorization to use the registry data.

## Results

### CRC mortality and its trends in Chinese cancer registration areas

From 2005 to 2018, there were 410,807 CRC deaths in cancer registration areas in China, including 237,890 males (57.90%) and 172,881 females (42.10%). There were 259,013 deaths (63.06%) in urban areas and 157,782 deaths (36.94%) in rural areas. The overall mortality rate was 14.45/100,000 (males 15.40/100,000, females 12.00/100,000; urban 16.31/100,000, rural 9.74/100,000), and the ASMR was 11.13/100,000 (95%CI: 11.03, 11.27) (males 13.38/100,000, females 9.10/100,000; urban 12.47/100,000, rural 8.72/100,000). The results showed that the mortality rate and ASMR of CRC in males were higher than those in females. The CRC mortality and ASMR in urban areas were higher than those in rural areas. (Table 3, Tables in S1 File)

**Table 3:** Mortality rate of CRC in Chinese cancer registration areas from 2005 to 2018 (/100,000).

Years	National			Male			Female			Urban			Rural		
	Deaths	Rates	ASMR (95%CI)	Deaths	Rates	ASMR (95%CI)	Deaths	Rates	ASMR (95%CI)	Deaths	Rates	ASMR (95%CI)	Deaths	Rates	ASMR (95%CI)
2005	7,049	12.83	11.40 (11.08, 11.62)	3,877	13.93	13.49 (13.00, 13.86)	3,172	11.71	9.60 (9.22, 9.90)	5,901	14.51	12.23 (12.10, 12.45)	1,149	8.07	8.45 (8.35, 8.56)
2006	7,979	13.40	11.78 (11.47, 11.99)	4,349	14.49	13.87 (13.38, 14.21)	3,632	12.29	10.06 (9.68, 10.34)	6,934	14.89	12.51 (12.38, 12.61)	1,044	8.03	8.46 (8.30, 8.57)
2007	8,475	14.17	12.19 (11.88, 12.40)	4,711	15.58	14.50 (14.02, 14.85)	3,754	12.69	10.14 (9.76, 10.42)	7,128	15.98	12.97 (12.87, 13.10)	1,344	8.84	9.19 (9.09, 9.28)
2008	9,800	14.82	11.84 (11.55, 12.02)	5,213	15.64	13.52 (13.09, 13.82)	4,585	13.98	10.39 (10.04, 10.65)	8,575	16.44	12.64 (12.45, 12.80)	1,224	8.75	8.17 (8.05, 8.30)
2009	12,163	14.23	11.83 (11.56, 11.98)	6,801	15.73	14.05 (13.64, 14.31)	5,361	12.69	9.85 (9.53, 10.06)	9,819	17.08	13.51 (13.41, 13.70)	2,527	9.03	8.62 (8.54, 8.71)
2010	16,679	23.52	11.30 (11.07, 11.42)	9,492	15.05	13.70 (13.36, 13.91)	7,186	11.67	9.19 (9.09, 10.49)	12,757	15.95	12.85 (12.65, 12.98)	3,921	8.78	8.09 (8.00, 8.20)
2011	19,746	13.55	11.31 (11.10, 11.41)	11,297	15.34	13.74 (13.42, 13.93)	8,446	11.71	9.13 (8.88, 9.28)	14,404	16.46	13.01 (12.88, 13.11)	5,172	9.66	8.98 (8.90, 9.07)
2012	25,553	12.90	10.68 (10.50, 10.76)	14,546	14.49	12.86 (12.59, 13.00)	11,003	11.26	8.70 (8.49, 8.82)	16,247	16.17	12.29 (12.15, 12.41)	9,300	9.53	8.63 (8.51, 8.71)
2013	29,502	13.03	10.52 (10.34, 10.59)	16,794	14.62	12.63 (12.37, 12.75)	12,712	11.39	8.60 (8.41, 8.71)	18,363	16.45	12.22 (12.02, 12.44)	11,142	9.70	8.53 (8.45, 8.60)
2014	38,268	13.28	10.50 (10.35, 10.56)	22,116	15.13	12.72 (12.49, 12.83)	16,150	11.37	8.46 (8.29, 8.55)	23,745	16.48	12.12 (12.00, 12.41)	14,518	10.07	8.61 (8.55, 8.70)
2015	44,362	13.82	10.60 (10.45, 10.65)	25,950	15.94	12.99 (12.77, 13.09)	18,427	11.65	8.39 (8.09, 8.97)	26,360	17.10	12.26 (12.06, 12.50)	18,004	10.79	8.83 (8.73, 8.91)
2016	53,811	14.10	10.64 (10.50, 10.69)	31,552	16.30	13.05 (12.84, 13.13)	22,243	11.84	8.40 (8.25, 8.47)	32,750	17.00	12.16 (11.99, 12.31)	21,072	11.15	8.91 (8.85, 8.99)
2017	61,451	14.08	10.53 (10.39, 10.56)	36,203	16.37	12.99 (12.79, 13.06)	25,235	11.73	8.25 (8.10, 8.31)	35,838	16.81	11.93 (11.87, 12.06)	25,598	11.47	9.03 (8.96, 9.10)
2018	75,969	14.52	10.65 (10.52, 10.68)	44,989	16.95	13.25 (13.05, 13.3)	30,975	12.02	8.26 (8.12, 8.31)	40,192	17.03	11.89 (11.75, 12.06)	35,767	12.46	9.54 (9.44, 9.60)
Total	410,807	14.45	11.13 (11.03, 11.27)	237,890	15.40	13.38 (13.05, 13.61)	172,881	12.00	9.10 (8.97, 9.15)	259,013	16.31	12.47 (12.27, 12.60)	151,782	9.74	8.72 (8.65, 8.78)

CRC, colorectal cancer; ASMR, age-standardized mortality rate.

**Fig 1:**
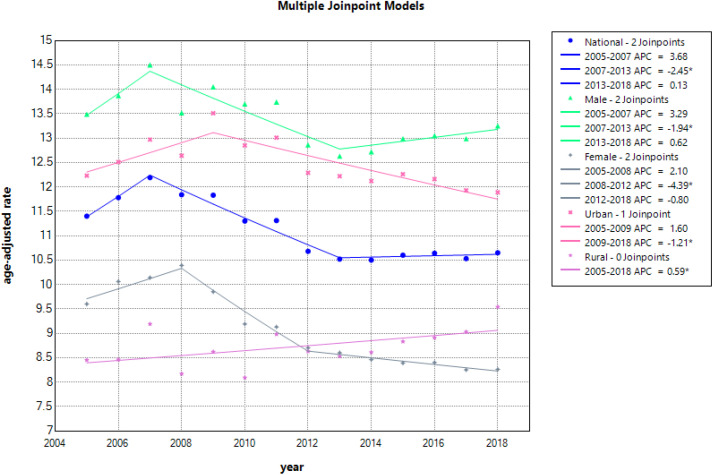
Joinpoint regression analysis of CRC mortality in Chinese cancer registration areas from 2005 to 2018. CRC, colorectal cancer; APC, annual percentage change.

The ASMR of Chinese CRC showed an upward trend from 2005 to 2007, with an APC of 3.67% (95% CI: −0.77%, 8.33%); from 2007 to 2013, there was a significant downward trend with an APC of −2.45% (95% CI: −3.14%, −1.47%); from 2013 to 2018, there was an upward trend with an APC of 0.13% (95% CI: −0.88%, 1.17%). The trends of ASMR for male and female CRC patients during the period from 2005 to 2018 were consistent with those of the entire country. In terms of urban-rural differences, from 2009 to 2018, the CRC ASMR in urban areas showed showed a downward trend, with an APC of −1.21% (95% CI: −1.71%, −0.71%), while in rural areas, it showed a significant upward trend, with an AAPC of 0.59% (95% CI: 0.24, 1.56%). (Table 4, Fig 1).

**Table 4:** Trend in CRC mortality in Chinese cancer registration areas from 2005 to 2018 (%).

Indexes	National	Male	Female	Urban	Rural
Year	2005-2007	2005-2007	2005-2007	2005-2009	2005-2018
APC (95%CI)	3.67 (−0.77, 8.33)	3.29 (−4.38, 11.58)	2.01 (−1.23, 4.38)	1.60 (−1.71, 3.39)	0.59^*^ (0.24, 1.56)
Year	2007-2013	2007-2013	2007-2013	2009-2018	
APC (95%CI)	−2.45^*^ (−3.14, −1.47)	−1.94^*^ (−3.64, −0.21)	−4.38^*^ (−6.51, −2.21)	−1.21^*^ (−1.71, −0.71)	
Year	2013-2018	2013-2018	2013-2018		
APC (95%CI)	0.13 (−0.88, 1.17)	0.62 (−1.14, 2.42)	−0.80 (−1.60, 0.01)		
AAPC (95%CI)	−0.53 (−1.25, 0.19)	−0.17 (−1.43, 1.11)	−1.27^*^ (−2.00, −0.52)	−0.35 (−0.91, 0.20)	0.59^*^ (0.24, 1.56)

CRC, colorectal cancer; AAPC: average annual percentage change; APC: annual percentage change. *The difference is statistically significant, *P <* 0.05.

### Age-period-cohort analysis

Regarding the impact of age, from 2005 to 2018, the pattern of CRC mortality rates across different regions of China generally remained consistent with respect to age. In the 0–40 age group, the risk of CRC death increased slowly with age; after the age of 40, the risk increased rapidly, reaching its peak in the 80–84 age group.

**Fig 2:**
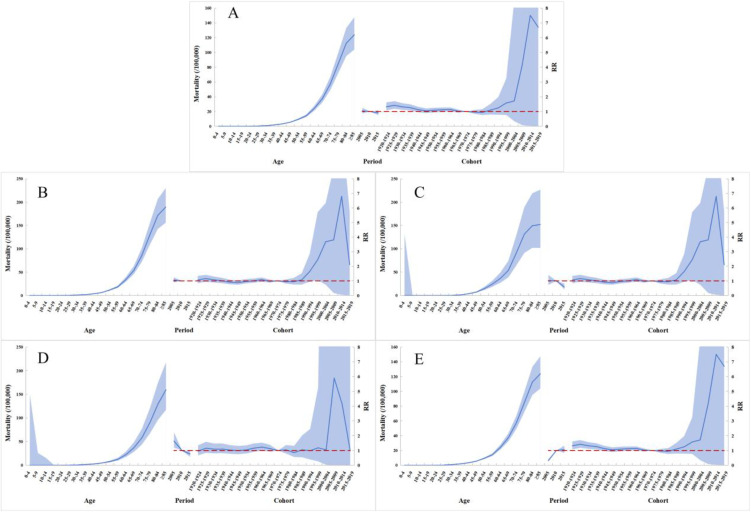
Age-period-cohort analysis of CRC mortality among nation (A), male (B), female (C), urban (D), and rural areas (E) in China from 2005 to 2018.

Regarding the cohort effects, the overall national and gender-specific CRC death risks began to rise from the 1980–1984 birth cohort and peaked in the 2010–2014 birth cohort. In urban areas, the CRC death risk started to increase rapidly from the 2000–2004 birth cohort and peaked in the 2010–2014 birth cohort. In rural areas, the death risk remained at a relatively high level, sharply increasing from the 1980–1984 birth cohort and peaking in the 2010–2014 birth cohort. (Fig 2).

### Prediction of CRC mortality in Chinese cancer registration areas

This study predicted the ASMR of CRC in Chinese cancer registration areas from 2019 to 2035 based on the BAPC model. The performance of the model was evaluated using multiple goodness-of-fit indicators (MSE, MAE, and MAPE). As shown in Table 5, all indicators showed good model fit, indicating that the BAPC model has high reliability and stability in predicting CRC ASMR nationally and across different subgroups (gender, urban-rural).

**Table 5:** Fitness of BAPC model from 2019 to 2035.

Dimension	MSE	MAE	MAPE (%)	Fitness
National	2.7*10^−8^	0.00016	0.05	99.94
Male	6.8*10^−8^	0.00026	0.06	99.97
Female	3.1*10^−8^	0.00017	0.05	99.98
Urban	5.9*10^−8^	0.00024	0.06	99.95
Rural	1.9*10^−8^	0.00014	0.01	99.98

BAPC, Bayesian age-period-cohort; MSE, mean squared error; MAE, mean absolute error; MAPE, mean absolute percentage error.

The prediction results indicated that from 2019 to 2035, the ASMR of CRC Chinese cancer registration areas will generally show an upward trend. The estimated ASMR for 2019, 2020, 2025, 2030, and 2035 were 10.34/100,000 (95%CI: 9.50, 11.19), 10.34/100,000 (95%CI: 9.08, 11.61), 10.41/100,000 (95%CI: 6.20, 14.62), 11.33/100,000 (95%CI: 2.20, 20.45), and 17.12/100,000 (95%CI: 0.19, 45.93), respectively.

The ASMR for males will show an upward trend from 2019 to 2035, while it will show a downward trend for females. For males, the ASMR was expected to increase from 12.93/100,000 (95%CI: 11.91, 13.94) in 2019 to 17.60/100,000 (95%CI: 0.15, 39.34) in 2035. During the same period, the ASMR of females decreased from 7.30/100,000 (95%CI: 7.17, 14.76) to 4.55 (95%CI: 0.70, 22.79).

**Fig 3:**
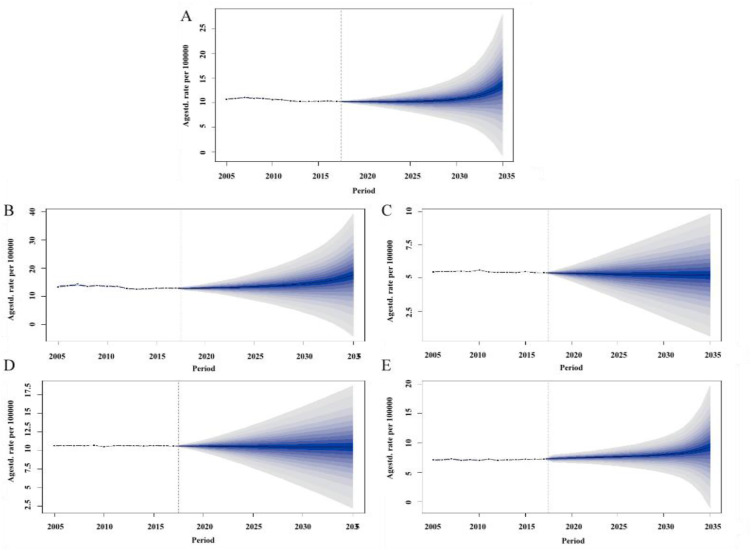
Prediction of CRC ASMR among nation (A), male (B), female (C), urban (D), and rural areas (E) in Chinese cancer registration areas from 2019 to 2035.ASMR, age-standardized mortality rate; CRC, colorectal cancer.

The ASMR in urban areas will decrease from 2019 to 2035, while in rural areas it will increase. The urban ASMR was predicted to decrease from 11.43/100,000 (95%CI: 11.23, 21.63) in 2019 to 9.15/100,000 (95%CI: 4.02, 28.33) in 2035. In rural areas, it was predicted to increase from 9.55/100,000 (95%CI: 6.65, 12.44) to 17.22/100,000 (95%CI: 0.35, 58.80), showing an upward trend. (Table 6, Fig 3)

**Table 6:** ASMR of CRC in Chinese cancer registration areas from 2019 to 2035 (/100,000).

Year	National		Male		Female		Urban		Rural	
	ASMR	95% CI	ASMR	95% CI	ASMR	95% CI	ASMR	95% CI	ASMR	95% CI
2019	10.34	9.50, 11.19	12.93	11.91, 13.94	7.30	7.17, 14.76	11.43	11.23, 21.63	9.55	6.65, 12.44
2020	10.34	9.08, 11.61	13.03	11.54, 14.51	7.02	6.87, 15.90	11.22	10.48, 21.92	9.74	6.47, 13.01
2021	10.35	8.61, 12.09	13.11	11.09, 15.13	6.75	6.29, 16.39	11.02	9.93, 22.97	9.92	6.19, 13.65
2022	10.32	8.06, 12.59	13.14	10.53, 15.76	6.50	5.81, 17.11	10.83	9.58, 22.64	10.09	5.82, 14.37
2023	10.31	7.46, 13.17	13.20	9.90, 16.49	6.27	5.45, 17.30	10.65	9.53,22.83	10.26	5.36, 15.17
2024	10.35	6.84, 13.85	13.33	9.28, 17.39	6.06	5.28, 17.99	10.49	9.50, 23.47	10.44	4.83, 16.06
2025	10.41	6.20, 14.62	13.48	8.61, 18.35	5.86	4.74, 18.06	10.32	8.83, 23.47	10.63	4.23, 17.03
2026	10.50	5.53, 15.47	13.63	7.89, 19.37	5.67	4.63, 19.17	10.17	8.47, 23.81	10.82	3.56, 18.08
2027	10.59	4.80, 16.38	13.75	7.08, 20.42	5.50	4.32, 19.32	10.02	8.42, 24.46	11.01	2.81, 19.22
2028	10.74	4.02, 17.47	13.92	6.22, 21.62	5.34	3.84, 19.51	9.89	7.67, 24.76	11.25	1.98, 20.52
2029	10.99	3.17, 18.81	14.18	5.32, 23.04	5.19	3.38, 19.76	9.77	7.19, 24.73	11.54	1.02, 22.05
2030	11.33	2.20, 20.45	14.47	4.33, 24.61	5.06	2.94, 20.06	9.65	6.91, 25.21	11.90	0.75, 23.94
2031	11.80	1.03, 22.57	14.80	3.23, 26.36	4.93	2.53, 20.40	9.53	6.85, 25.92	12.38	0.72, 26.47
2032	12.48	0.53, 25.48	15.20	1.97, 28.44	4.82	2.17, 20.81	9.43	5.03, 26.89	13.03	0.66, 30.12
2033	13.48	0.47, 29.74	15.77	0.54, 31.10	4.72	1.90, 21.34	9.33	4.48, 27.15	13.96	0.62, 35.74
2034	14.97	0.22, 36.17	16.57	0.41, 34.64	4.63	1.74, 22.00	9.24	4.17, 27.66	15.30	0.44, 44.64
2035	17.12	0.19, 45.93	17.60	0.15, 39.34	4.55	0.70, 22.79	9.15	4.02, 28.33	17.22	0.35, 58.80

ASMR, age-standardized mortality rate; CRC, colorectal cancer; 95% CI, 95% confidence interval.

**Fig 4:**
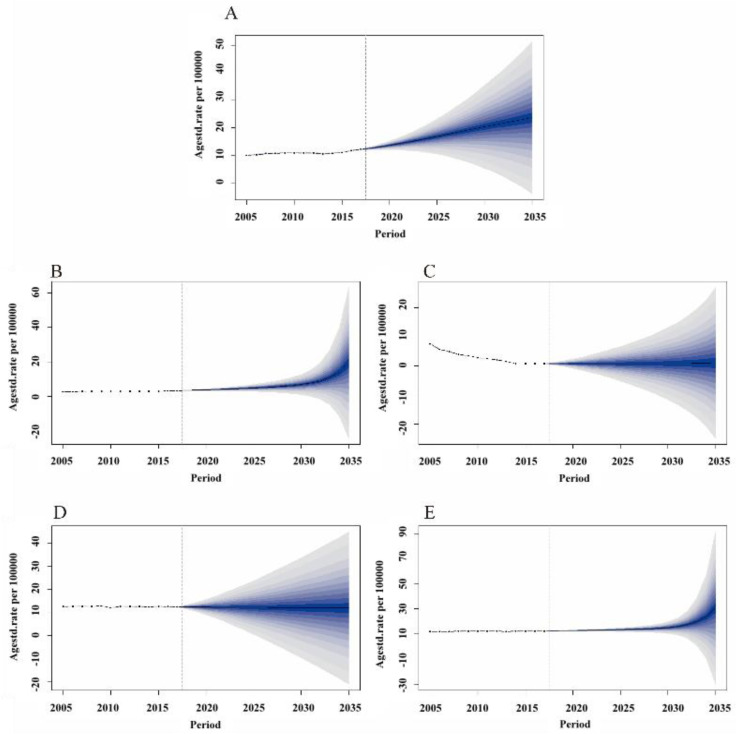
Predicted CRC ASMR in China for the overall population (A), males (B), females (C), urban areas (D), and rural areas (E), 2019–2035, based on GBD data.ASMR, age-standardized mortality rate; CRC, colorectal cancer.

To further verify the accuracy of the prediction results in this study, the BAPC model was used to analyze the CRC death data from GBD database from 2005 to 2018. The results showed a similar trend to the conclusions of this study. (Fig 4)

### Spatial distribution characteristics of CRC mortality in Chinese cancer registration areas

**Fig 5:**
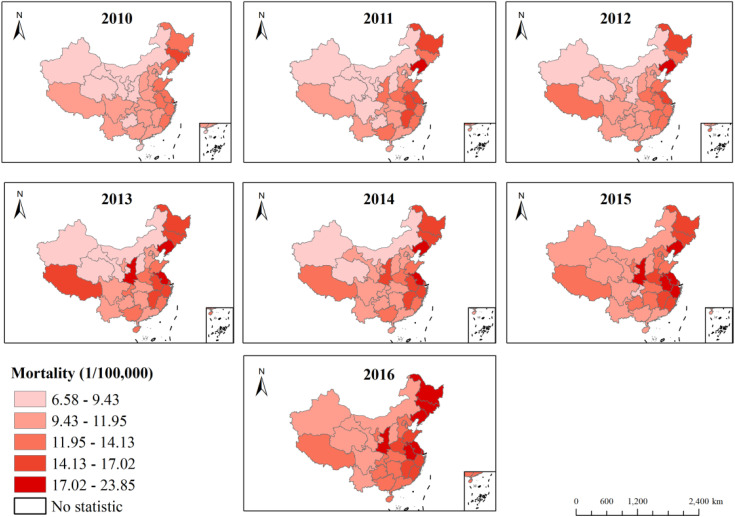
Mortality rate of CRC in Chinese cancer registration areas by region from 2010 to 2016. CRC, colorectal cancer (Map from the Ministry of Natural Resources of China, cartographic license: GS (2019) 1822).

Between 2010 and 2016, CRC mortality was higher in eastern coastal and northeastern regions in Chinese cancer registration areas, while mortality was relatively lower in western and northwestern regions. Specifically, in 2010, regions with higher mortality included Beijing (10.21/100,000), Tianjin (10.91/100,000), Shanghai (15.16/100,000), Jiangsu (13.76/100,000), and Zhejiang (13.52/100,000). By 2016, high mortality values were still concentrated in eastern coastal areas, such as Beijing (12.44/100,000), Tianjin (13.29/100,000), Shanghai (18.69/100,000), Jiangsu (19.48/100,000), and Zhejiang (16.89/100,000). In contrast, western and northwestern regions such as Xinjiang (10.21/100,000), Tibet (13.87/100,000), Gansu (10.78/100,000), and Qinghai (10.49/100,000) consistently had lower mortality. (Table 7, Fig 5)

**Table 7:** Mortality rate of CRC in Chinese cancer registration areas by region from 2010 to 2016 (/100,000).

Provinces	2010	2011	2012	2013	2014	2015	2016
Beijing	10.21	9.20	10.49	9.52	10.67	12.07	12.44
Tianjin	10.91	10.44	11.52	10.29	11.27	13.12	13.29
Hebei	10.38	9.44	10.78	9.92	10.88	12.45	12.67
Shanxi	9.67	8.58	9.94	8.85	9.90	11.33	11.42
Inner Mongolia	8.85	7.76	7.88	8.18	9.39	9.95	11.24
Liaoning	12.82	13.68	13.85	14.79	15.63	15.81	16.51
Jilin	14.46	13.01	13.99	14.84	14.51	16.91	17.84
Heilongjiang	13.33	15.54	16.22	15.83	15.17	16.58	19.22
Shanghai	15.16	14.84	14.95	15.24	14.35	14.13	18.69
Jiangsu	13.76	16.16	14.97	18.21	17.05	19.55	19.48
Zhejiang	13.52	13.78	13.94	15.02	14.77	17.23	16.89
Anhui	12.37	15.02	13.21	16.81	15.36	17.43	17.62
Fujian	12.75	12.45	12.88	12.92	13.16	15.03	14.85
Jiangxi	11.89	14.21	12.19	15.50	14.29	14.61	16.25
Shandong	12.67	12.63	12.36	13.59	12.94	13.69	14.47
Henan	10.80	12.05	13.53	12.80	13.16	15.60	15.18
Hubei	10.30	10.33	11.83	10.43	11.19	12.90	12.56
Hunan	10.23	10.16	11.53	10.47	10.91	12.12	12.09
Guangdong	10.77	10.60	11.74	11.65	11.30	11.65	12.37
Guangxi	10.12	12.01	11.06	13.62	12.23	11.17	13.39
Hainan	8.65	11.28	12.22	13.37	11.67	10.86	12.69
Chongqing	9.63	11.03	9.96	13.36	10.95	8.83	12.55
Sichuan	9.46	9.39	10.95	9.96	10.14	10.96	11.11
Guizhou	9.38	9.22	10.97	10.84	10.92	12.17	11.98
Yunnan	9.80	10.00	11.81	11.48	11.04	10.94	11.81
Shaanxi	9.32	12.92	9.26	17.75	16.27	18.83	18.44
Gansu	8.13	8.31	9.65	8.58	9.65	10.91	10.78
Qinghai	8.78	6.94	6.58	7.34	9.14	10.20	10.49
Ningxia	8.82	8.07	7.01	9.76	11.11	12.79	12.60
Xinjiang	8.46	7.48	8.24	7.69	9.15	10.17	10.21
Tibet	11.95	11.00	13.81	14.87	12.70	12.47	13.87

CRC, colorectal cancer.

Global spatial autocorrelation analysis of national CRC mortality from 2010 to 2016 showed significant positive spatial autocorrelation characteristics. The Moran’s I index for each year was greater than 0, and the *P*-value was less than 0.05, indicating that CRC mortality was clustered nationwide. Among these years, 2010 had the highest Moran’s *I* index (0.336), indicating the most pronounced spatial clustering of mortality that year. (Table 8)

**Table 8:** Global spatial autocorrelation analysis of CRC mortality in Chinese cancer registration areas from 2010 to 2016.

Years	Moran’s *I* index	Expected index	Variance	Z	*P*
2010	0.336	−0.033	0.006	4.845	0.000
2011	0.149	−0.033	0.005	2.559	0.010
2012	0.141	−0.033	0.004	2.495	0.013
2013	0.143	−0.033	0.006	2.306	0.021
2014	0.174	−0.033	0.006	2.723	0.006
2015	0.175	−0.033	0.006	2.759	0.006
2016	0.193	−0.033	0.006	2.978	0.003

CRC, colorectal cancer.

**Fig 6:**
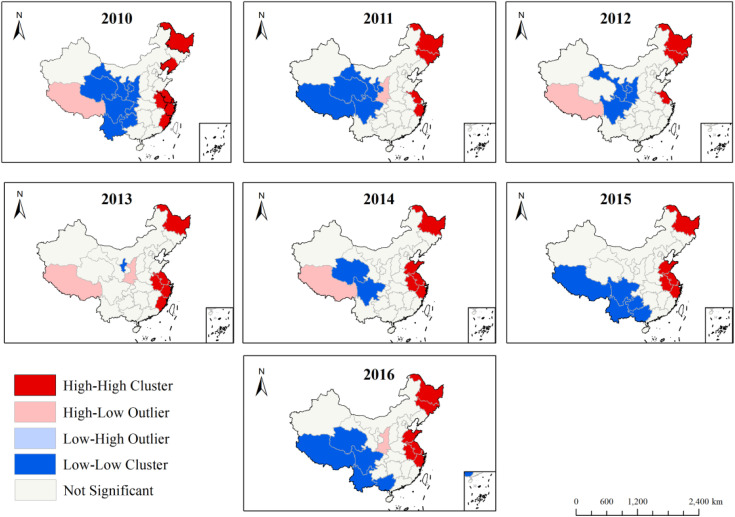
Local spatial autocorrelation of CRC mortality in Chinese cancer registration areas from 2010 to 2016. CRC, colorectal cancer (Map from the Ministry of Natural Resources of China, cartographic license: GS (2019) 1822).

Local spatial autocorrelation analysis further revealed significant spatial differentiation characteristics of high and low clustering areas of CRC mortality (Fig 6). Hotspot areas were mainly distributed in eastern coastal regions (such as Zhejiang, Jiangsu, and Shanghai) and northeastern regions (such as Heilongjiang, Jilin, and Liaoning), where the local Moran’s I index was positive. Coldspot areas were mainly located in central and western regions, such as Gansu, Ningxia, and Qinghai. From 2010 to 2016, hotspot areas remained consistently stable in eastern coastal and northeastern regions; while coldspot areas showed a trend of migrating southwestward, gradually shifting from regions like Gansu, Ningxia, and Qinghai to regions such as Tibet, Yunnan, and Guangxi.

## Discussion

This study was based on the China Cancer Registry data, systematically analyzing the long-term trends, age-period-cohort effects, and spatial distribution characteristics of CRC mortality in Chinese cancer registration areas. The results showed that the CRC mortality rate in Chinese cancer registration areas showed an upward-downward- upward trend from 2005 to 2018, and then continued to rise until 2035. At the same time, it still faces a series of challenges such as urban-rural, gender, and regional inequality.

From 2005 to 2007, the mortality of CRC in Chinese cancer registration areas showed an upward trend, which may reflect the comprehensive influence of multiple factors. Studies have clearly indicated that excessive intake of red and processed meat, as well as unhealthy lifestyles such as smoking and alcohol consumption, are important modifiable risk factors for CRC incidence and mortality [33]. There is evidence to suggest that since the late 1980s, with the rapid development of the Chinese economy and the acceleration of urbanization, residents’ lifestyles have gradually become “westernized,” accompanied by significant changes in dietary structure and physical activity levels. Firstly, there has been a significant increase in meat intake, with per capita meat consumption rapidly rising from about 4 kg/year in the 1960s to about 70 kg/year in 2022, an increase of nearly 18 times, with a significant increase in red and processed meat intake. At the same time, the dietary structure shifted towards high-fat and high energy density, with daily energy intake increasing from approximately 2,330 kcal in 1980–2,940 kcal in 2002. In this context, the prevalence of overweight and obesity has significantly increased. Study showed that the overweight and obesity rate among Chinese adults increased from 12.9% in 1991 to 27.3% in 2004. Some studies have also found that abdominal obesity increased from 17.9% to 42.5% (males) between 1993 and 2006 [34]. In addition, the level of physical activity has significantly decreased, and the proportion of people engaged in heavy physical labor has decreased from 65% in 1989 to 51% in 2000 [35]. At the same time, unhealthy behaviors are still quite common, for example, there are currently about 300 million smokers in China, with a male smoking rate of nearly 50%. Alcohol consumption and high-fat eating habits have also increased with economic development [36]. These dietary structure changes, the prevalence of obesity, and widespread unhealthy behaviors may jointly contribute to the increase in CRC mortality.

From 2007 to 2013, the ASMR of CRC in Chinese cancer registration areas showed a significant downward trend. Since 2005, the CRC screening program in China has gradually been promoted nationwide, and the application of key screening methods such as colonoscopy has been increasing, especially in urban areas. Studies have shown that in population-based screening programs in China, the compliance rate of high-risk individuals with colonoscopy has gradually increased from about 14% in the early stages to over 20%−40%, and can even reach 42.5% in different regions [37]. In addition, the multi provincial joint screening studies have included hundreds of thousands to millions of subjects, further promoting the detection of early lesions [38]. A study has shown that screening can effectively increase the detection rate of early (stage I-II) CRC and reduce the risk of mortality [39]. In addition, at the public level, health awareness and early medical behavior have gradually improved. Although early studies have shown that the awareness level of CRC screening among Chinese residents is relatively low, with the strengthening of health education and public health interventions, the population’s awareness and willingness to participate in screening have significantly increased, providing an important foundation for the implementation of screening programs for CRC [40,41].

However, after entering the plateau period of prevention and control strategies in 2013, the downward trend tended to flatten out. The coverage of urban screening is facing bottlenecks, and the marginal cost and difficulty of further expanding coverage and increasing the participation rate of high-risk groups have increased [42]. Meanwhile, the prevalence of risk factors such as overweight, obesity, and sedentary lifestyle among the general population has not been effectively curbed [43]. In addition, low levels of education and inadequate access to healthcare are significantly associated with reduced participation in CRC screening, with these factors being more concentrated in rural areas [44,45]. A study has shown that the likelihood of rural populations undergoing CRC screening is significantly lower than that of urban populations (OR=0.81, 95%CI: 0.76–0.86), indicating that urban-rural differences still exist [46]. These factors may have led to a resurgence in the mortality rate of CRC.

Age-period-cohort analysis showed that the risk of death from CRC significantly increased with age, accelerating after the age of 40 and peaking in the 80–84 age group, which was consistent with known biological mechanisms [47]. The continuous intensification of population aging has increased the number of high-risk individuals and the overall burden of CRC [48]. Cohort effect analysis showed that the estimated risk of CRC death had been on the rise since the birth cohort in 1980–1984, and the estimated value for recent birth cohorts was even higher. Population and nutrition monitoring data showed that late birth cohorts were generally more exposed to several known risk factors, such as a significant increase in per capita meat and processed meat consumption, rising rates of overweight/obesity, and long-term high male smoking rates. These generational exposure differences may partially explain the estimated cohort effects [49–51]. However, it should be noted that individuals born between 2010 and 2014 are currently young and have limited follow-up time. Therefore, in these recently born cohorts, estimates of “high risk” should be interpreted with caution and require long-term follow-up and exposure outcome studies to confirm.

Spatial analysis showed that there was a significant positive spatial autocorrelation in the mortality rate of CRC in Chinese cancer registration areas, with hotspots consistently concentrated in the eastern coastal and northeastern regions. In contrast, areas with lower mortality rates were mainly distributed in the central and western regions. However, economically developed regions typically have more complete cancer registration and diagnostic resources, with higher detection rates for cases and deaths, thereby amplifying observed hotspots. In contrast, less developed regions have relatively lower medical accessibility, as well as lower endoscopic diagnostic capabilities and coverage of early CRC screening. This may result in a large number of cases not being effectively recorded and reported, leading to an artificially underestimated death statistics. Future study should incorporate registration quality indicators and screening/medical resource indicators, and conduct joint modeling with regional incidence rates and socioeconomic variables, in order to more accurately distinguish “real risks” from “monitoring errors.”

Our predictions indicated that between 2019 and 2035, the ASMR of CRC in Chinese cancer registration areas may increase overall, with different patterns based on gender and place of residence: men’s predicted ASMR continued to rise, while women’s predicted ASMR decreased; urban ASMR was showing a downward trend, while rural ASMR was expected to increase. The challenges in the prevention and control of CRC in China still exist: progress in urban areas may reflect wider acceptance of screening, diagnosis, and treatment capabilities, as well as increased public awareness, while growth in rural areas may reflect limited access to screening and diagnostic services, lower early detection rates, and continued changes in lifestyle and risk factor exposure. The increase in male specificity may partially reflect lower screening participation rates and higher exposure to behavioral risk factors. These predictions highlight the priority of action-strengthening rural screening and diagnostic capabilities, incorporating CRC screening into primary and community health services, and designing outreach and prevention programs for men and other high-risk groups.

It is important to acknowledge the limitations of this study. The data are sourced from cancer registration areas, which do not fully cover the entire national population, and the expanding coverage may have an impact on the analysis of time trends, especially the early trends, resulting in a certain degree of conservativeness in the overall results. There is a reporting delay of approximately three years in cancer registration data, which affects the timeliness of the results of this study. The provincial-level data used for spatial analysis are only from the China Cancer Registry Annual Reports from 2013 to 2019, which limits the temporal scope of the spatial analysis. Furthermore, there are regional disparities in registration coverage and data quality across China. More developed regions often possess superior medical resources and information systems, have established cancer registration systems earlier, and possess stronger case confirmation capabilities, which may lead to relatively higher reported mortality rates. Additionally, key confounding variables, including age structure, urbanization level, and registration quality, are not available, thus making it impossible to adjust for them in the analysis. Future study should incorporate standardized mortality indicators, registration quality indicators, and socioeconomic factors, and apply more advanced spatial analysis methods to more comprehensively assess the mortality patterns of CRC.

## Conclusion

The overall mortality of CRC in Chinese cancer registration areas showed a fluctuating trend from 2005 to 2018, and was expected to increase from 2019 to 2035. The mortality in rural areas continued to rise, and the risk of male remained higher than that of females. In addition, the eastern coastal and northeastern regions showed a clear spatial trend of high incidence clustering. The prevention and control of CRC still face multiple challenges of inequality between urban and rural areas, gender, and regions. To achieve CRC prevention and control, it is necessary to strengthen the early screening and diagnosis capabilities in rural areas, carry out dietary and lifestyle interventions for high-risk areas, improve the quality and comprehensive coverage of national cancer registration data, and comprehensively enhance the level of CRC prevention and control to reduce its burden.

## Supporting information

S1 File All supplementary tables are provided in S1 File. (XLSX)

## References

[1] Bizuayehu HM, Ahmed KY, Kibret GD, Dadi AF, Belachew SA, Bagade T. Global disparities of cancer and its projected burden in 2050. Jama Netw Open. 2024;7(11):e2443198. doi: 10.1001/jamanetworkopen.2024.43198 PMC11539015 39499513

[2] Cancer today. https://gco.iarc.who.int/media/globocan/factsheets/cancers/39-all-cancers-fact-sheet.pdf. Accessed 2025 August 29.

[3] Cancer tomorrow. https://gco.iarc.who.int/today/. Accessed 2025 August 29.

[4] Gbd results. https://vizhub.healthdata.org/gbd-results. Accessed 2025 August 29.

[5] Xi Y, Xu P. Global colorectal cancer burden in 2020 and projections to 2040. Transl Oncol. 2021;14(10):101174. doi: 10.1016/j.tranon.2021.101174 34243011 PMC8273208

[6] Zhang Z, Zhang L, Liu Y, Roy M, Lu P, Zhang M. Interpretation of the 2022 global cancer statistics report. Chinese Hospital Statistics. 2024;31(05):393–400. doi: 10.3969/j.issn.1006-5253.2024.05.014

[7] Chen W, Zhao P. 2008 China cancer registry annual report. Beijing: Military Medical Science Press. 2009.

[8] Chen W, Zhao P. China cancer registry annual report. Beijing: Military Medical Science Press. 2010.

[9] Chen W, Zhao P. China cancer registry annual report. Beijing: Military Medical Science Press. 2011.

[10] Chen W, He J i e, Zhao P i n g. China cancer registry annual report. Beijing: Military Medical Science Press. 2012.

[11] Chen W, He J. 2012 China cancer registry annual report. Beijing: Military Medical Science Press. 2013.

[12] Chen W, He J. China cancer registry annual report. Beijing: Tsinghua University Press. 2014.

[13] Chen W, He J. 2014 China cancer registry annual report. Beijing: Tsinghua University Press. 2015.

[14] Chen W, He J. China cancer registry annual report. Beijing: Tsinghua University Press. 2016.

[15] Chen W, He J. 2016 China cancer registry annual report. Beijing: Tsinghua University Press; 2017.

[16] Chen W, He J. 2017 China cancer registry annual report. Beijing: People’s Medical Publishing House. 2018.

[17] He J. China cancer registry annual report. Beijing: People’s Medical Publishing House. 2019.

[18] Wei W, He J. China cancer registry annual report. Beijing: People’s Medical Publishing House. 2020.

[19] Wei W, He J. 2020 China cancer registry annual report. Beijing: People’s Medical Publishing House. 2021.

[20] Wenqiang W, Jie H. 2021 China cancer registry annual report. Beijing: People’s Medical Publishing House; 2022.

[21] National Bureau of Statistics. Census data. https://www.stats.gov.cn/sj/pcsj/

[22] Outland B, Newman MM, William MJ. Health Policy Basics: Implementation of the International Classification of Disease, 10th Revision. Ann Intern Med. 2015;163(7):554–6. doi: 10.7326/M15-1933 26390305

[23] Fay MP, Feuer EJ. Confidence intervals for directly standardized rates: a method based on the gamma distribution. Stat Med. 1997;16(7):791–801. doi: 10.1002/(sici)1097-0258(19970415)16:7<791::aid-sim500>3.0.co;2-# 9131766

[24] Lin K, Jia H, Cao M, Xu T, Chen Z, Song X, et al. Epidemiological characteristics of leukemia in China, 2005-2017: a log-linear regression and age-period-cohort analysis. BMC Public Health. 2023;23(1):1647. doi: 10.1186/s12889-023-16226-1 37641011 PMC10464264

[25] Lin K, Shao J, Cao Y, Lu L, Lei P, Chen X, et al. The trend of lymphoma incidence in China from 2005 to 2017 and lymphoma incidence trend prediction from 2018 to 2035: a log-linear regression and Bayesian age-period-cohort analysis. Front Oncol. 2024;14:1297405. doi: 10.3389/fonc.2024.1297405 38868533 PMC11167089

[26] Liu X. Applicability evaluation of endogenous estimators and construction and application of Bayesian models in age period queue analysis. Huazhong University of Science and Technology. 2022.

[27] Zheng R, Chen W. Introduction to age period queue prediction model based on Bayesian method. Chinese Journal of Preventive Medicine. 2012;46(7):648–50. doi: 10.3760/cma.j.issn.0253-9624.2012.07.016

[28] Shi Z, Shao J, Dong C, Song G, Hu Y, Niu Q, et al. Burden of chronic obstructive pulmonary disease attributable to non-optimal temperature, 1990-2044: six countries on the same isotherm. BMC Public Health. 2024;24(1):3407. doi: 10.1186/s12889-024-20622-6 39695480 PMC11653645

[29] Github - timcdlucas/inlautils: R package providing utilities for inla. https://github.com/timcdlucas/INLAutils. Accessed 2025 November 7.

[30] Bayesian age-period-cohort model. https://github.com/uniwander/BayesianAgePeriodCohort. 2025.

[31] Chen Y. New approaches for calculating Moran’s index of spatial autocorrelation. PLoS One. 2013;8(7):e68336. doi: 10.1371/journal.pone.0068336 23874592 PMC3709922

[32] National Cancer Prevention and Control Platform. https://www.chinancpcn.org.cn/home. Accessed 2025 November 19.

[33] Johnson CM, Wei C, Ensor JE, Smolenski DJ, Amos CI, Levin B, et al. Meta-analyses of colorectal cancer risk factors. Cancer Causes Control. 2013;24(6):1207–22. doi: 10.1007/s10552-013-0201-5 23563998 PMC4161278

[34] Over the last six decades, China has rapidly increased and diversified its meat consumption. https://ourworldindata.org/data-insights/over-the-last-six-decades-china-has-rapidly-increased-and-diversified-its-meat-consumption. Accessed 2026 March 24.

[35] Wang Z, Zhang B, Zhai F, Wang H, Zhang J, Du W, et al. Fatty and lean red meat consumption in China: differential association with Chinese abdominal obesity. Nutr Metab Cardiovasc Dis. 2014;24(8):869–76. doi: 10.1016/j.numecd.2014.03.002 24795160 PMC4112159

[36] In China TWCOTROCHAD, Hu S-S. Report on cardiovascular health and diseases in China 2021: an updated summary. J Geriatr Cardiol. 2023;20(6):399–430. doi: 10.26599/1671-5411.2023.06.001 37416519 PMC10320777

[37] Chen H, Lu B, Dai M. Colorectal cancer screening in china: status, challenges, and prospects - china, 2022. China Cdc Wkly. 2022;4(15):322–8. doi: 10.46234/ccdcw2022.077 35548454 PMC9081894

[38] Zhang J, Xu H, Zheng L, Yu J, Chen Q, Cao X, et al. Determinants of Participation and Detection Rate of Colorectal Cancer From a Population-Based Screening Program in China. Front Oncol. 2020;10:1173. doi: 10.3389/fonc.2020.01173 32850337 PMC7412959

[39] Lin G, Feng Z, Liu H, Li Y, Nie Y, Liang Y. Mass screening for colorectal cancer in a population of two million older adults in Guangzhou, China. Sci Rep. 2019;9(1):10424. doi: 10.1038/s41598-019-46670-2 31320661 PMC6639356

[40] Huang R-L, Liu Q, Wang Y-X, Zou J-Y, Hu L-F, Wang W, et al. Awareness, attitude and barriers of colorectal cancer screening among high-risk populations in China: a cross-sectional study. BMJ Open. 2021;11(7):e045168. doi: 10.1136/bmjopen-2020-045168 34253663 PMC8276297

[41] Yu Z, Li B, Zhao S, Du J, Zhang Y, Liu X, et al. Uptake and detection rate of colorectal cancer screening with colonoscopy in China: A population-based, prospective cohort study. Int J Nurs Stud. 2024;153:104728. doi: 10.1016/j.ijnurstu.2024.104728 38461798

[42] Liu M, Huang S, Yu Z, Dai L, Xiang J, Qu Y, et al. Assessing factors influencing participation in LDCT lung cancer screening among high-risk urban populations in Nanjing, China. BMC Cancer. 2025;25(1):1196. doi: 10.1186/s12885-025-14589-9 40691785 PMC12278565

[43] Farvid MS, Sidahmed E, Spence ND, Mante Angua K, Rosner BA, Barnett JB. Consumption of red meat and processed meat and cancer incidence: a systematic review and meta-analysis of prospective studies. Eur J Epidemiol. 2021;36(9):937–51. doi: 10.1007/s10654-021-00741-9 34455534

[44] Leung DYP, Chow KM, Lo SWS, So WKW, Chan CWH. Contributing factors to colorectal cancer screening among Chinese people: a review of quantitative studies. Int J Environ Res Public Health. 2016;13(5). doi: 10.3390/ijerph13050506 PMC4881131 27196920

[45] Zhang X, Yang L, Liu S, Li H, Li Q, Li H, et al. Performance of different colorectal cancer screening strategies: a long-term passive follow-up population-based screening program in Beijing, China. BMC Public Health. 2023;23(1):1640. doi: 10.1186/s12889-023-16564-0 37641033 PMC10463986

[46] Sepassi A, Li M, Zell JA, Chan A, Saunders IM, Mukamel DB. Rural-urban disparities in colorectal cancer screening, diagnosis, treatment, and survivorship care: a systematic review and meta-analysis. Oncologist. 2024;29(4):e431-46. doi: 10.1093/oncolo/oyad347 PMC10994268 38243853

[47] Jiang T-J, Wang F, Wang Y-N, Hu J-J, Ding P-R, Lin J-Z, et al. Germline mutational profile of Chinese patients under 70 years old with colorectal cancer. Cancer Commun (Lond). 2020;40(11):620–32. doi: 10.1002/cac2.12093 32914570 PMC7668457

[48] Luo S, Gong J, Zhu Y, Wang L, Zhang K. Global, regional, and national burden of colorectal cancer in the elderly (aged > 60 years): a comprehensive analysis across 204 countries and territories (1990-2021). BMC Gastroenterol. 2025;25(1):570. doi: 10.1186/s12876-025-04184-4 40783503 PMC12335148

[49] Li Y, Wang DD, Ley SH, Howard AG, He Y, Lu Y, et al. Potential Impact of Time Trend of Life-Style Factors on Cardiovascular Disease Burden in China. J Am Coll Cardiol. 2016;68(8):818–33. doi: 10.1016/j.jacc.2016.06.011 27539174 PMC5850940

[50] Liu S, Zhang M, Yang L, Li Y, Wang L, Huang Z, et al. Prevalence and patterns of tobacco smoking among Chinese adult men and women: findings of the 2010 national smoking survey. J Epidemiol Community Health. 2017;71(2):154–61. doi: 10.1136/jech-2016-207805 27660401 PMC5284482

[51] Global, regional, and national prevalence of adult overweight and obesity, 1990-2021, with forecasts to 2050: a forecasting study for the global burden of disease study 2021. Lancet. 2025;405(10481):813–38. doi: 10.1016/S0140-6736(25)00355-1 40049186 PMC11920007

