# Supplementary material for: Spatio-temporal patterns and prediction of colorectal cancer mortality in Chinese cancer registration areas: A nationwide study based on multiple models
Source: PLoS One. 2026 Jul 2;21(7):e0352110. doi: 10.1371/journal.pone.0352110 (PMC13327207; doi:10.1371/journal.pone.0352110)
Supplement: Attachment — Submitted filename: Response to Reviewers.docx [file pone.0352110.s003.docx]

PLOS ONE

March 26, 2026

PONE-D-25-66196

"Spatio-temporal patterns and prediction of colorectal cancer mortality in Chinese cancer registration areas: A nationwide study based on multiple models"

**Dear Editor and Reviewers:**

We are very grateful to receive comments on the revision of the manuscript entitled "Spatio-temporal patterns and prediction of colorectal cancer mortality in Chinese cancer registration areas: A nationwide study based on multiple models" (ID: PONE-D-25-66196).

We sincerely thank all editors and reviewers for their valuable feedback that we have used to clarify the thinking of the study, make the structure of the study more reasonable, and finally improve the quality of our manuscript.

On the separate pages, we provided our response to the comments and suggestions, point by point, and highlighted the changes in the marked copy of the revision. We hope that our revision will be approved by the experts and reviewed favorably.

Sincerely,

Jiageng He

**Reviewer Comments:**

**Reviewer 1**

The manuscript addresses an important topic—spatiotemporal patterns of CRC mortality in China and prediction of future trends—using registry report data (2005–2018) and multiple analytic approaches (Joinpoint regression, age–period–cohort models, Bayesian APC projection, and spatial autocorrelation). The overall direction of the descriptive findings (age gradients, geographic clustering, and differences by sex and urban/rural strata) is plausible.

**Response:** Thank you very much for your recognition and support. We have made revisions to the professional issues, requirements and suggestions you pointed out, and have also improved the entire text. We hope to continue to receive your recognition and professional guidance.

*1. Registry coverage increased markedly over the study period, which may influence the observed temporal trends. Please report the population coverage and number of registries contributing data for each year, and clarify whether the reported rates reflect registry areas only or are intended to represent national estimates. A sensitivity analysis restricted to registries with continuous reporting across the study period would help assess the robustness of the Joinpoint results.*

**Response:** We sincerely appreciate these valuable comments. During the course of this study, we supplemented data on the number of registry sites, population size, coverage, and quality control from cancer registry areas in China. The data used in this study were derived from the China Cancer Registry Annual Report. Although the coverage is not 100%, this study includes comprehensive regions across eastern, central, and western China, and currently represents the most reliable data source for reflecting the national cancer epidemiological profile. Since the mortality rates mentioned in the annual report are only those of the cancer registry areas, to ensure greater accuracy and a more cautious expression, the authors have restricted the geographic description throughout the manuscript to “Chinese cancer registry areas.” Of course, this is also one of the limitations of this study. We have mentioned the limitation in the section on limitations. We sincerely hope for your understanding and approval.

The revised content has been highlighted in blue.

In lines 85-110:

**Data quality**

To ensure the authenticity, stability, and comparability of the data, data collection, verification, and analysis were conducted in accordance with the Chinese Cancer Registry Guidelines and the standards of the IARC, ensuring consistency in registration procedures and coding practices across regions and study years. All registries underwent standardized evaluations of comparability, completeness, and validity.

The inclusion and exclusion of data strictly adhered to the quality control standards for Chinese cancer registration work. Data included in the report must meet the following core quality requirements: the morphological verification percentage (MV%) should range between 55% and 95%; the percentage of death certificate only (DCO%) should be less than 20%; the mortality-to-incidence ratio (M/I) should fall within the range of 0.55 to 0.85.

From 2008 to 2021, the coverage of cancer registries reported in the China Cancer Registry Annual Report expanded steadily. It increased from 45 cancer registries in 2005, covering a population of 69,369,668 (accounting for 5.31% of the national population at the end of 2005), to 947 registries by 2018, covering 634,376,540 individuals (representing 45.14% of the national population at the end of 2018) (Table 1). From 2005 to 2018, the MV%, DCO%, and the M/I were 67.83%, 2.11%, and 0.62, respectively, all of which met national quality benchmarks (Table 2). These indicators collectively demonstrate that the registry data are robust and reliable, providing a sound foundation for epidemiological analysis.

**Table 1 Number of cancer registries and population coverage in China, 2005–2018**

| Year | Number of cancer registries | Covered population | Population coverage (%) |
| --- | --- | --- | --- |
| 2005 | 45 | 69,369,668 | 5.31 |
| 2006 | 49 | 76,209,748 | 5.80 |
| 2007 | 48 | 70,782,375 | 5.36 |
| 2008 | 56 | 82,433,497 | 6.21 |
| 2009 | 104 | 109,476,347 | 8.20 |
| 2010 | 219 | 207,229,403 | 15.42 |
| 2011 | 234 | 221,390,275 | 16.43 |
| 2012 | 261 | 239,887,749 | 17.64 |
| 2013 | 347 | 287,284,044 | 21.11 |
| 2014 | 449 | 345,711,646 | 25.27 |
| 2015 | 501 | 387,872,825 | 28.22 |
| 2016 | 682 | 476,692,113 | 34.47 |
| 2017 | 821 | 563,934,185 | 40.57 |
| 2018 | 947 | 634,376,540 | 45.14 |

**Table 2 Quality indicators of cancer registry data in China, 2005–2018**

| Year | National | | |  | Urban | | |  | Rural | | |
| --- | --- | --- | --- | --- | --- | --- | --- | --- | --- | --- | --- |
|  | MV(%) | DCO(%) | M/I |  | MV(%) | DCO(%) | M/I |  | MV(%) | DCO(%) | M/I |
| 2005 | 66.00 | 1.76 | 0.65 |  | 69.02 | 2.08 | 0.65 |  | 56.09 | 0.71 | 0.28 |
| 2006 | 65.59 | 1.66 | 0.64 |  | 67.71 | 1.85 | 0.61 |  | 57.11 | 0.90 | 0.76 |
| 2007 | 65.83 | 1.95 | 0.64 |  | 67.71 | 2.28 | 0.61 |  | 59.57 | 0.83 | 0.75 |
| 2008 | 69.33 | 2.23 | 0.62 |  | 70.53 | 2.49 | 0.59 |  | 64.22 | 1.12 | 0.73 |
| 2009 | 67.23 | 3.14 | 0.63 |  | 68.96 | 3.03 | 0.60 |  | 62.91 | 3.43 | 0.71 |
| 2010 | 67.11 | 2.99 | 0.61 |  | 71.51 | 2.49 | 0.59 |  | 60.65 | 3.72 | 0.64 |
| 2011 | 70.14 | 2.44 | 0.63 |  | 72.92 | 2.17 | 0.61 |  | 65.34 | 2.90 | 0.67 |
| 2012 | 69.13 | 2.38 | 0.62 |  | 70.63 | 2.63 | 0.59 |  | 67.31 | 2.09 | 0.65 |
| 2013 | 68.04 | 1.74 | 0.62 |  | 70.76 | 1.76 | 0.59 |  | 64.97 | 1.72 | 0.65 |
| 2014 | 68.01 | 2.91 | 0.61 |  | 69.75 | 2.79 | 0.58 |  | 65.96 | 1.49 | 0.64 |
| 2015 | 39.31 | 2.08 | 0.61 |  | 64.45 | 3.25 | 0.53 |  | 62.32 | 2.92 | 0.63 |
| 2016 | 68.31 | 1.40 | 0.61 |  | 70.03 | 1.58 | 0.59 |  | 66.29 | 1.18 | 0.63 |
| 2017 | 67.77 | 1.53 | 0.59 |  | 69.97 | 1.62 | 0.56 |  | 65.47 | 1.44 | 0.62 |
| 2018 | 69.94 | 1.34 | 0.58 |  | 71.50 | 1.58 | 0.55 |  | 68.48 | 1.11 | 0.61 |
| AVG | 65.84 | 2.11 | 0.62 |  | 69.68 | 2.26 | 0.59 |  | 63.34 | 1.83 | 0.64 |

MV%, morphological verification percentage; DCO%, percentage of death certificate only; M/I, mortality-to-incidence ratio.

*2. The methods section would benefit from greater detail on how mortality rates and ASMR were calculated. Please specify the age groupings used, the standardization approach (e.g., direct standardization), and whether provincial and urban–rural results are crude or age-standardized. Where feasible, provide uncertainty estimates for annual ASMR values, not only for APC/AAPC.*

**Response:** We sincerely appreciate the valuable comments. We have added the calculation formula for ASMR in the method. When analyzing 31 provincial-level administrative regions, only crude mortality rates were used due to data limitations. All other methods used age-standardized mortality rates for calculation, and we also supplemented the 95% confidence interval of ASMR as suggested.

The revised content has been highlighted in blue.

In lines 116-140:

**Statistical indicators**

Mortality rate, also known as crude mortality rate, refers to the number of cancer deaths registered per 100,000 population in a given year, and reflects the mortality level of the population. Since the crude mortality rate is significantly affected by the age composition of the population, when comparing and analyzing mortality rates across different regions or the mortality levels of the same population group over different periods, it is necessary to calculate the age-standardized mortality rate (ASMR) to eliminate the impact of population age structure on mortality. The ASMR refers to the mortality rate calculated according to the age structure of a standard population. In this study, mortality data across the country, different genders, and different regions were divided into 18 age groups with an interval of 5 years: 0-4 years, 5-9 years, 10-14 years...80-84 years, and 85 years and above. The standard population data came from the sixth national population census data released by the National Bureau of Statistics of China in 2010[21]. In this study, ASMR were used in the Joinpoint regression and BAPC models to ensure comparability over time and across populations. For provincial-level spatial analysis, crude mortality rates were used due to the lack of age-specific data in China Cancer Registry Annual Reports.

$$\text{Mortality}\text{ rate per 100,000=}\frac{\text{count}}{\text{population}}\text{×100,000}$$

$$\text{ASMR=}\frac{\sum\text{standard population in corresponding age group × age-specific mortality rate}}{\sum\text{standard population}}\text{×100,000}$$

The 95% confidence interval (CI) for the ASMR was estimated using the Fay-Feuer method, which is robust for directly standardized rates across varying population sizes and incidence levels[23].

$$\text{ASMR 95\%C}\text{I}_{\text{low}}\text{=}\left( \frac{\text{v}}{\text{2×ASMR}} \right)\text{×}\left( \text{ChiInv}\left( \frac{\text{0.05}}{\text{2}}\text{,}\frac{\text{(2×}\text{ASMR}^{\text{2}}\text{)}}{\text{v}} \right) \right)\text{×100,000}$$

$$\text{ASMR 95\%C}\text{I}_{\text{high}}\text{=}\left( \frac{\text{v+}\text{w}_{\text{m}}^{\text{2}}}{\text{2×(ASMR+}\text{w}_{\text{m}}\text{)}} \right)\text{×}\left( \text{ChiInv}\left( \text{1-}\frac{\text{0.05}}{\text{2}}\text{,}\frac{\text{2(ASMR+}\text{w}_{\text{m}}\text{)}^{\text{2}}}{\text{v+}\text{w}_{\text{m}}^{\text{2}}} \right) \right)\text{×100,000}$$

Where, $\text{w}_{\text{m}}$ is the weight correction factor, $\text{v}$ is the estimated variance of the ASMR, ChiInv (p, n) is the inverse of the chi-squared distribution function evaluated at p and with n degrees of freedom.

| **Table 3 Mortality rate of CRC in Chinese cancer registration areas from 2005 to 2018 (/100,000)** | | | | | | | | | | | | | | | | | | | |
| --- | --- | --- | --- | --- | --- | --- | --- | --- | --- | --- | --- | --- | --- | --- | --- | --- | --- | --- | --- |
| Years | National | | |  | Male | | |  | Female | | |  | Urban | | |  | Rural | | |
|  | Deaths | Rates | ASMR  (95%CI) |  | Deaths | Rates | ASMR  (95%CI) |  | Deaths | Rates | ASMR  (95%CI) |  | Deaths | Rates | ASMR  (95%CI) |  | Deaths | Rates | ASMR  (95%CI) |
| 2005 | 7,049 | 12.83 | 11.40  (11.08, 11.62) |  | 3,877 | 13.93 | 13.49  (13.00, 13.86) |  | 3,172 | 11.71 | 9.60  (9.22, 9.90) |  | 5,901 | 14.51 | 12.23  (12.10, 12.45) |  | 1,149 | 8.07 | 8.45  (8.35, 8.56) |
| 2006 | 7,979 | 13.40 | 11.78  (11.47, 11.99) |  | 4,349 | 14.49 | 13.87  (13.38, 14.21) |  | 3,632 | 12.29 | 10.06  (9.68, 10.34) |  | 6,934 | 14.89 | 12.51  (12.38, 12.61) |  | 1,044 | 8.03 | 8.46  (8.30, 8.57) |
| 2007 | 8,475 | 14.17 | 12.19  (11.88, 12.40) |  | 4,711 | 15.58 | 14.50  (14.02, 14.85) |  | 3,754 | 12.69 | 10.14  (9.76, 10.42) |  | 7,128 | 15.98 | 12.97  (12.87, 13.10) |  | 1,344 | 8.84 | 9.19  (9.09, 9.28) |
| 2008 | 9,800 | 14.82 | 11.84  (11.55, 12.02) |  | 5,213 | 15.64 | 13.52  (13.09, 13.82) |  | 4,585 | 13.98 | 10.39  (10.04, 10.65) |  | 8,575 | 16.44 | 12.64  (12.45, 12.80) |  | 1,224 | 8.75 | 8.17  (8.05, 8.30) |
| 2009 | 12,163 | 14.23 | 11.83  (11.56, 11.98) |  | 6,801 | 15.73 | 14.05  (13.64, 14.31) |  | 5,361 | 12.69 | 9.85  (9.53, 10.06) |  | 9,819 | 17.08 | 13.51  (13.41, 13.70) |  | 2,527 | 9.03 | 8.62  (8.54, 8.71) |
| 2010 | 16,679 | 23.52 | 11.30  (11.07, 11.42) |  | 9,492 | 15.05 | 13.70  (13.36, 13.91) |  | 7,186 | 11.67 | 9.19  (9.09, 10.49) |  | 12,757 | 15.95 | 12.85  (12.65, 12.98) |  | 3,921 | 8.78 | 8.09  (8.00, 8.20) |
| 2011 | 19,746 | 13.55 | 11.31  (11.10, 11.41) |  | 11,297 | 15.34 | 13.74  (13.42, 13.93) |  | 8,446 | 11.71 | 9.13  (8.88, 9.28) |  | 14,404 | 16.46 | 13.01  (12.88, 13.11) |  | 5,172 | 9.66 | 8.98  (8.90, 9.07) |
| 2012 | 25,553 | 12.90 | 10.68  (10.50, 10.76) |  | 14,546 | 14.49 | 12.86  (12.59, 13.00) |  | 11,003 | 11.26 | 8.70  (8.49, 8.82) |  | 16,247 | 16.17 | 12.29  (12.15, 12.41) |  | 9,300 | 9.53 | 8.63  (8.51, 8.71) |
| 2013 | 29,502 | 13.03 | 10.52  (10.34, 10.59) |  | 16,794 | 14.62 | 12.63  (12.37, 12.75) |  | 12,712 | 11.39 | 8.60  (8.41, 8.71) |  | 18,363 | 16.45 | 12.22  (12.02, 12.44) |  | 11,142 | 9.70 | 8.53  (8.45, 8.60) |
| 2014 | 38,268 | 13.28 | 10.50  (10.35, 10.56) |  | 22,116 | 15.13 | 12.72  (12.49, 12.83) |  | 16,150 | 11.37 | 8.46  (8.29, 8.55) |  | 23,745 | 16.48 | 12.12  (12.00, 12.41) |  | 14,518 | 10.07 | 8.61  (8.55, 8.70) |
| 2015 | 44,362 | 13.82 | 10.60  (10.45, 10.65) |  | 25,950 | 15.94 | 12.99  (12.77, 13.09) |  | 18,427 | 11.65 | 8.39  (8.09, 8.97) |  | 26,360 | 17.10 | 12.26  (12.06, 12.50) |  | 18,004 | 10.79 | 8.83  (8.73, 8.91) |
| 2016 | 53,811 | 14.10 | 10.64  (10.50, 10.69) |  | 31,552 | 16.30 | 13.05  (12.84, 13.13) |  | 22,243 | 11.84 | 8.40  (8.25, 8.47) |  | 32,750 | 17.00 | 12.16  (11.99, 12.31) |  | 21,072 | 11.15 | 8.91  (8.85, 8.99) |
| 2017 | 61,451 | 14.08 | 10.53  (10.39, 10.56) |  | 36,203 | 16.37 | 12.99  (12.79, 13.06) |  | 25,235 | 11.73 | 8.25  (8.10, 8.31) |  | 35,838 | 16.81 | 11.93  (11.87, 12.06) |  | 25,598 | 11.47 | 9.03  (8.96, 9.10) |
| 2018 | 75,969 | 14.52 | 10.65  (10.52, 10.68) |  | 44,989 | 16.95 | 13.25  (13.05, 13.3) |  | 30,975 | 12.02 | 8.26  (8.12, 8.31) |  | 40,192 | 17.03 | 11.89  (11.75, 12.06) |  | 35,767 | 12.46 | 9.54  (9.44, 9.60) |
| Total | 410,807 | 14.45 | 11.13  (11.03, 11.27) |  | 237,890 | 15.40 | 13.38  (13.05, 13.61) |  | 172,881 | 12.00 | 9.10  (8.97, 9.15) |  | 259,013 | 16.31 | 12.47  (12.27, 12.60) |  | 151,782 | 9.74 | 8.72  (8.65, 8.78) |

CRC, colorectal cancer; ASMR, age-standardized mortality rate.

*3. Additional information is needed on Joinpoint model settings, including the maximum number of joinpoints allowed, the model selection procedure, and the final joinpoint locations for each subgroup. This will improve transparency and reproducibility.*

**Response:** We sincerely appreciate the valuable comments. In this study, Joinpoint regression analysis was conducted using the Joinpoint regression program. The maximum number of joinpoints is set to 4. Model selection is performed using Monte Carlo permutation test, which is the default and recommended method in Joinpoint software. The final number and location of connection points for each subgroup are automatically determined by the model selection process described above. We have supplemented it in the methods, The revised content has been highlighted in blue.

In lines 142-166:

**Joinpoint regression model**

Joinpoint regression was applied to analyze temporal trends in CRC mortality. This method fits segmented regression models to time-series data and identifies points where statistically significant changes in trends occur. Given that cancer mortality data typically follow a Poisson or exponential distribution, a log-linear Joinpoint model was employed in this study[24, 25]. The model can be expressed as:

$$\text{ln}\left( \text{R}_{\text{y}} \right)\text{=}\text{β}_{\text{0}}\text{+}\text{β}_{\text{1}}\text{y}\text{+}\sum_{\text{k}\text{=1}}^{\text{K}} \text{δ}_{\text{k}}\left( \text{y}\text{−}\text{τ}_{\text{k}} \right)^{\text{+}}$$

Where, $\text{R}_{\text{y}}$ denotes the CRC mortality (per 100,000 population) in year y, $\text{β}_{\text{0}}$ is the intercept, $\text{β}_{\text{1}}$ is the baseline slope, $\text{τ}_{\text{k}}$ represents the location of the k-th joinpoint, and ($\text{y}\text{−}\text{τ}_{\text{k}}$)+=max(0, $\text{y}\text{−}\text{τ}_{\text{k}}$). The number of joinpoints (K) was set to four in this study.

Model selection was performed using the Monte Carlo permutation test, which identifies the optimal number of joinpoints by comparing goodness-of-fit across models.

The period 2005–2018 was divided into multiple segments. The annual percentage change (APC) was used to evaluate within-segment trends, while the average annual percentage change (AAPC) described the overall trend. A 95% CI was used to assess statistical significance. When the number of joinpoints equals zero, APC=AAPC. APC>0 or AAPC>0 indicates an increasing trend, while APC<0 or AAPC<0 indicates a decreasing trend.

APC calculation formula:

$\text{APC=}\left( \text{e}^{\text{β}_{\text{1}}}\text{−1} \right)\text{×}$100

AAPC calculation formula:

$\text{AAPC=}\left[ \text{exp}\left( \sum\text{ω}_{\text{i}}\text{β}_{\text{i}}\text{/}\sum\text{ω}_{\text{i}} \right)\text{−1} \right]\text{×}$100

In the formula, $\text{β}_{\text{1}}$ is the regression coefficient, $\text{β}_{\text{i}}$ is the regression coefficient corresponding to each interval and $\text{ω}_{\text{i}}$ is the span of each interval (i.e. the number of years included in the interval).

*4. The choice of intrinsic estimator should be briefly justified, with clear description of the age, period, and cohort intervals. The very wide prediction intervals by 2035 suggest substantial model uncertainty; out-of-sample validation or presentation of shorter-term projections as primary results would strengthen confidence in the forecasts. Interpretations of cohort and period effects should also be phrased cautiously given the ecological design.*

**Response:** We sincerely appreciate the valuable comments. We provided a more detailed explanation of the age-period-cohort and BAPC model, and to ensure the accuracy and reliability of the results, we conducted sensitivity analysis using GBD data from the same period. The results also yielded similar conclusions to our structure.

The revised content has been highlighted in blue.

In lines 167-191:

**Age–period–cohort model**

The age–period–cohort model is based on the Poisson distribution and is used to identify the independent effects of age, period, and cohort on observed variables[26]. The model is expressed as:

$$\log\left( \text{λ}_{\text{apc}} \right)\text{=}\text{α}_{\text{a}}\text{+}\text{β}_{\text{p}}\text{+}\text{γ}_{\text{c}}\text{+}\text{ε}$$

In the formula, α、β and γ represent the effects of age, period, and cohort, while ε represents the residual.

According to the requirements of the age-period-cohort model, the data needs to be organized into the following format:

Age group

This study included data on CRC deaths in various age groups from 2005 to 2018. The CRC death cases in China were divided into 18 age groups, ranging from 0-4 years, 5-9 years...up to 85 years and above, based on the age group of every 5 years.

Period group

In order to comply with the norms of the age-period-cohort model, this study divided the time span from 2005 to 2018 into periods every 5 years, resulting in a total of 3 periods: 2005-2009, 2010-2014, and 2015-2018.

Cohort group

In the age-period-cohort model, the birth cohort was obtained by subtracting age from period. Therefore, this study defined 20 birth cohorts from 1920-1924 to 2015-2019.

The results of age, period, and cohort effect are usually measured by relative risk (RR) to assess changes in the risk of death in different age groups, periods, or birth cohorts relative to the reference group, where RR>1 indicates increased risk and RR<1 indicates decreased risk.

In lines 192-233:

**Bayesian age–period–cohort model**

To address the identifiability problem in the traditional age–period–cohort model— arising from the exact linear dependency among age, period, and cohort—this study employed a BAPC model to perform smoothed estimation and predict the long-term trend of CRC mortality. The BAPC model incorporates Bayesian random effects and smoothing priors within the age–period–cohort framework, providing stable parameter estimates while preserving the interpretability of the three temporal factors[27, 28]. Unlike the intrinsic estimator (IE) approach commonly used in traditional age–period–cohort analysis, the BAPC model addresses the identifiability problem through Bayesian smoothing priors (e.g., second-order random walk priors), which impose structural constraints on adjacent parameters. This approach allows for stable and interpretable estimation of age, period, and cohort effects without requiring explicit identification restrictions.

Model specification

Let $\text{Y}_{\text{ap}}\text{ }$denote the number of CRC cases in age group a and period p, and $\text{E}_{\text{ap}}$ denote the corresponding population at risk. We assumed:

$$\text{Y}_{\text{ap}}\text{∼Poisson}\left( \text{E}_{\text{ap}}\text{λ}_{\text{ap}} \right)$$

Where, $\text{λ}_{\text{ap}}$ represents the mortality rate for the age–period unit.

The log-linear form of the model is expressed as:

$$\text{log}\left( \text{λ}_{\text{ap}} \right)\text{=μ+}\text{α}_{\text{a}}\text{+}\text{β}_{\text{p}}\text{+}\text{γ}_{\text{c}}$$

Where, μ is the intercept, $\text{α}_{\text{a}}$, $\text{β}_{\text{p}}$ and $\text{γ}_{\text{c}}$ denote the age, period, and cohort effects, respectively, and c=p-a.

Prior distributions

To ensure model smoothness and identifiability, the age, period, and cohort effects were assigned second-order random walk (RW2) priors:

$$\text{p}\left( \text{α|}\text{k}_{\text{α}} \right)\text{∝}\text{k}_{\text{α}}^{\left( \text{I−2} \right)\text{/2}}\text{exp}\left( \text{−}\frac{\text{k}_{\text{α}}}{\text{2}}\sum_{\text{i=3}}^{\text{I}} \left( \text{α}_{\text{i}}\text{−2}\text{α}_{\text{i−1}}\text{+}\text{α}_{\text{i−2}} \right)^{\text{2}} \right)$$

$$\text{p}\left( \text{β|}\text{k}_{\text{β}} \right)\text{∝}\text{k}_{\text{β}}^{\left( \text{J−2} \right)\text{/2}}\text{exp}\left( \text{−}\frac{\text{k}_{\text{β}}}{\text{2}}\sum_{\text{j=3}}^{\text{J}} \left( \text{β}_{\text{j}}\text{−2}\text{β}_{\text{j−1}}\text{+}\text{β}_{\text{j−2}} \right)^{\text{2}} \right)$$

$$\text{p}\left( \text{γ|}\text{k}_{\text{γ}} \right)\text{∝}\text{k}_{\text{γ}}^{\left( \text{K−2} \right)\text{/2}}\text{exp}\left( \text{−}\frac{\text{k}_{\text{γ}}}{\text{2}}\sum_{\text{k=3}}^{\text{K}} \left( \text{γ}_{\text{k}}\text{−2}\text{γ}_{\text{k−1}}\text{+}\text{γ}_{\text{k−2}} \right)^{\text{2}} \right)$$

Here, $\text{k}_{\text{α}}$, $\text{k}_{\text{β}}$, and $\text{k}_{\text{γ}}$ are the precision parameters controlling the smoothness of age, period, and cohort effects, respectively. To reflect weakly informative prior assumptions, their hyperpriors were defined as:

$$\text{k}_{\text{α}}\text{,}\text{k}_{\text{β}}\text{,}\text{k}_{\text{γ}}\text{∼Gamma}\left( \text{1,0.0005} \right)$$

This prior structure is widely applied in BAPC modeling and effectively balances over-smoothing and overfitting.

Model implementation and posterior inference

The model was implemented in R 4.4.0 using the BAPC and INLA (Integrated Nested Laplace Approximation) packages[29, 30]. The INLA algorithm was applied to approximate posterior distributions and to obtain the posterior means and 95%CI for the parameters.

In this study, the BAPC model was used to predict the ASMR of CRC in China from 2019 to 2035, stratified by sex and region. The ages were categorized into consecutive 5-year groups, which was consistent with the standard practices in age–period–cohort analysis. Model fit was evaluated using mean squared error (MSE), mean absolute error (MAE), and mean absolute percentage error (MAPE).

In addition, to assess the robustness and predictive performance of the BAPC model, a sensitivity analysis was conducted using historical data. Specifically, ASMR of CRC in China from the Global Burden Of Disease (GBD) study during 2005–2018 were used for validation.

In lines 335-337:

To further verify the accuracy of the prediction results in this study, the BAPC model was used to analyze the CRC death data from GBD database from 2005 to 2018. The results showed a similar trend to the conclusions of this study. (Fig 4)

**Fig 4 Predicted CRC ASMR in China for the overall population (A), males (B), females (C), urban areas (D), and rural areas (E), 2019–2035, based on GBD data. ASMR, age-standardized mortality rate; CRC, colorectal cancer.**

*5. The manuscript requires substantial English language editing for clarity and grammar, and several sentences over-interpret associations (diet, screening, microbiome) not directly tested in this ecological analysis. Please revise the discussion to frame these as hypotheses consistent with prior literature and expand limitations accordingly.*

**Response:** We sincerely appreciate the valuable comments. We have reviewed and revised the language of the entire text. We have modified the discussion section, focusing more on describing the changes in our research results rather than explaining their mechanism of action. We hope to receive your understanding and support.

**Reviewer 2:**

This is a methodologically ambitious, data-rich descriptive epidemiology study suitable for a broad journal audience. Its main strengths lie in comprehensive national surveillance synthesis and pattern visualization. However, the manuscript would benefit from tighter epidemiologic framing, clearer acknowledgment of ecological and surveillance limitations, and more cautious interpretation of APC and predictive findings.

**Response:** Thank you very much for your careful review and professional guidance. We are very glad to have received your approval.

*1. Morans I is good but you didnt adjust for confoundsrs. Such as age structure, urbanization, or registry quality.*

**Response:** We sincerely appreciate your scientific suggestions. We agree that spatial clustering may be influenced by potential confounding factors, such as age structure, urbanization level, and registration quality. However, due to the limitations of the data in China Cancer Registration Annual Report, key confounding variables (including age structure, urbanization level, and registration quality) in each registration area cannot be obtained, and thus cannot be adjusted in the analysis. We have provided additional information on this in the methods section and limitations section.

The revised content has been highlighted in blue.

In lines 131-132:

For provincial-level spatial analysis, crude mortality rates were used due to the lack of age-specific data in China Cancer Registry Annual Reports.

In lines 503-520:

It is important to acknowledge the limitations of this study. The data are sourced from cancer registration areas, which do not fully cover the entire national population, and the expanding coverage may have an impact on the analysis of time trends, especially the early trends, resulting in a certain degree of conservativeness in the overall results. There is a reporting delay of approximately three years in cancer registration data, which affects the timeliness of the results of this study. The provincial-level data used for spatial analysis are only from the China Cancer Registry Annual Reports from 2013 to 2019, which limits the temporal scope of the spatial analysis. Furthermore, there are regional disparities in registration coverage and data quality across China. More developed regions often possess superior medical resources and information systems, have established cancer registration systems earlier, and possess stronger case confirmation capabilities, which may lead to relatively higher reported mortality rates. Additionally, key confounding variables, including age structure, urbanization level, and registration quality, are not available, thus making it impossible to adjust for them in the analysis. Future study should incorporate standardized mortality indicators, registration quality indicators, and socioeconomic factors, and apply more advanced spatial analysis methods to more comprehensively assess the mortality patterns of CRC.

*2. Hotspot regions may reflect better case ascertainment, not higher underlying risk. Spatial findings may conflate surveillance intensity with disease burden.*

**Response:** We sincerely appreciate the valuable comments. We have revised the discussion section to acknowledge that regions with more developed healthcare systems and higher quality cancer registration may achieve more complete case capture and reporting, which may lead to higher observed mortality rates. Therefore, the observed spatial clustering may reflect the true disease burden and differences in monitoring intensity in different regions. In addition, we have also supplemented this with limitations

The revised content has been highlighted in blue.

In lines 478-487:

However, economically developed regions typically have more complete cancer registration and diagnostic resources, with higher detection rates for cases and deaths, thereby amplifying observed hotspots. In contrast, less developed regions have relatively lower medical accessibility, as well as lower endoscopic diagnostic capabilities and coverage of early CRC screening. This may result in a large number of cases not being effectively recorded and reported, leading to an artificially underestimated death statistics. Future study should incorporate registration quality indicators and screening/medical resource indicators, and conduct joint modeling with regional incidence rates and socioeconomic variables, in order to more accurately distinguish "real risks" from "monitoring errors".

In lines 503-520:

It is important to acknowledge the limitations of this study. The data are sourced from cancer registration areas, which do not fully cover the entire national population, and the expanding coverage may have an impact on the analysis of time trends, especially the early trends, resulting in a certain degree of conservativeness in the overall results. There is a reporting delay of approximately three years in cancer registration data, which affects the timeliness of the results of this study. The provincial-level data used for spatial analysis are only from the China Cancer Registry Annual Reports from 2013 to 2019, which limits the temporal scope of the spatial analysis. Furthermore, there are regional disparities in registration coverage and data quality across China. More developed regions often possess superior medical resources and information systems, have established cancer registration systems earlier, and possess stronger case confirmation capabilities, which may lead to relatively higher reported mortality rates. Additionally, key confounding variables, including age structure, urbanization level, and registration quality, are not available, thus making it impossible to adjust for them in the analysis. Future study should incorporate standardized mortality indicators, registration quality indicators, and socioeconomic factors, and apply more advanced spatial analysis methods to more comprehensively assess the mortality patterns of CRC.

*3. Forecast uncertainty becomes extremely wide after ~2030, with implausibly large confidence intervals (e.g., ASMR CI spanning near zero to >40). Can you do sensitivity analyses (e.g., alternative priors, truncated forecast horizons) as long range predictions are not reliable.*

**Response:** We sincerely appreciate your careful review. We fully agree with your viewpoint. Not only did we use some indicators to reflect the stability of the model and the reliability of the prediction results, but also to ensure the accuracy of the results, we conducted a sensitivity analysis using GBD CRC mortality data from 2005 to 2018, and the results were generally similar to ours. We hope our modifications can gain your support and approval.

The revised content has been highlighted in blue.

In lines 230-233:

In addition, to assess the robustness and predictive performance of the BAPC model, a sensitivity analysis was conducted using historical data. Specifically, ASMR of CRC in China from the Global Burden Of Disease (GBD) study during 2005–2018 were used for validation.

In lines 335-337:

To further verify the accuracy of the prediction results in this study, the BAPC model was used to analyze the CRC death data from GBD database from 2005 to 2018. The results showed a similar trend to the conclusions of this study. (Fig 4)

**Fig 4 Predicted CRC ASMR in China for the overall population (A), males (B), females (C), urban areas (D), and rural areas (E), 2019–2035, based on GBD data. ASMR, age-standardized mortality rate; CRC, colorectal cancer.**

*4. Cohort effects peaking in 2010–2014 birth cohorts are difficult to interpret epidemiologically, given limited follow-up time for these cohorts. Period effects (e.g., screening expansion, healthcare reforms) are discussed but not empirically evaluated.*

**Response:** We sincerely appreciate the valuable comments. We have supplemented the discussion section. We hope our modifications can gain your support and approval. The revised content has been highlighted in blue.

In lines 462-473:

Cohort effect analysis showed that the estimated risk of CRC death had been on the rise since the birth cohort in 1980-1984, and the estimated value for recent birth cohorts was even higher. Population and nutrition monitoring data showed that late birth cohorts were generally more exposed to several known risk factors, such as a significant increase in per capita meat and processed meat consumption, rising rates of overweight/obesity, and long-term high male smoking rates. These generational exposure differences may partially explain the estimated cohort effects[49-51]. However, it should be noted that individuals born between 2010 and 2014 are currently young and have limited follow-up time. Therefore, in these recently born cohorts, estimates of "high risk" should be interpreted with caution and require long-term follow-up and exposure outcome studies to confirm.

*5. Cancer registry coverage expanded substantially over time (~49% population coverage), which may bias temporal trends, especially early increases. Apparent trends may partially reflect surveillance artifacts rather than true mortality changes. Add limitations or correct interpretations*

**Response:** We sincerely appreciate the valuable comments. We have supplemented it with limitations. The revised content has been highlighted in blue.

In lines 503-520:

It is important to acknowledge the limitations of this study. The data are sourced from cancer registration areas, which do not fully cover the entire national population, and the expanding coverage may have an impact on the analysis of time trends, especially the early trends, resulting in a certain degree of conservativeness in the overall results. There is a reporting delay of approximately three years in cancer registration data, which affects the timeliness of the results of this study. The provincial-level data used for spatial analysis are only from the China Cancer Registry Annual Reports from 2013 to 2019, which limits the temporal scope of the spatial analysis. Furthermore, there are regional disparities in registration coverage and data quality across China. More developed regions often possess superior medical resources and information systems, have established cancer registration systems earlier, and possess stronger case confirmation capabilities, which may lead to relatively higher reported mortality rates. Additionally, key confounding variables, including age structure, urbanization level, and registration quality, are not available, thus making it impossible to adjust for them in the analysis. Future study should incorporate standardized mortality indicators, registration quality indicators, and socioeconomic factors, and apply more advanced spatial analysis methods to more comprehensively assess the mortality patterns of CRC.

*6. My major concenrn is risk of ecological fallacy and over-interpretation of cohort and spatial effects. The study is purely descriptive and ecological, yet portions of the Discussion imply causal interpretations (e.g., diet, screening effects, Westernization). APC and BAPC results should be framed as pattern decomposition and projection, not mechanisms.*

**Resonse:** We sincerely appreciate the valuable comments. We have changed the discussion section to focus more on describing the changes in our results rather than interpreting the mechanisms of action. We hope to obtain your understanding and support.
