# Supplementary material for: Spatio-temporal patterns and prediction of colorectal cancer mortality in Chinese cancer registration areas: A nationwide study based on multiple models
Source: PLoS One. 2026 Jul 2;21(7):e0352110. doi: 10.1371/journal.pone.0352110 (PMC13327207; doi:10.1371/journal.pone.0352110)
Supplement: Peer review history

# Peer review: Decision Letter 0

**DOI:** 10.1371/journal.pone.0352110.r001

**Authors:** Ana Paula Drummond Lage

11 Mar 2026

PONE-D-25-66196Spatio-temporal patterns and prediction of colorectal cancer mortality in Chinese Cancer Registration Areas: A nationwide study based on multiple modelsPLOS One

Dear Dr. He,

Thank you for submitting your manuscript to PLOS ONE. After careful consideration, we feel that it has merit but does not fully meet PLOS ONE’s publication criteria as it currently stands. Therefore, we invite you to submit a revised version of the manuscript that addresses the points raised during the review process.

==============================

ACADEMIC EDITOR:

The reviewers suggest major revisions to your manuscript.  Therefore, I invite you to respond to the reviewer's comments, point-by-point, and revise your manuscript.

Please submit your revised manuscript by 03/10/2026. If you will need more time than this to complete your revisions, please reply to this message or contact the journal office at plosone@plos.org. When you're ready to submit your revision, log on to https://www.editorialmanager.com/pone/ and select the 'Submissions Needing Revision' folder to locate your manuscript file.

Please include the following items when submitting your revised manuscript:

- A letter that responds to each point raised by the academic editor and reviewer(s). You should upload this letter as a separate file labeled 'Response to Reviewers'.
- A marked-up copy of your manuscript that highlights changes made to the original version. You should upload this as a separate file labeled 'Revised Manuscript with Track Changes'.
- An unmarked version of your revised paper without tracked changes. You should upload this as a separate file labeled 'Manuscript'.

If you would like to make changes to your financial disclosure, please include your updated statement in your cover letter. Guidelines for resubmitting your figure files are available below the reviewer comments at the end of this letter.

If applicable, we recommend that you deposit your laboratory protocols in protocols.io to enhance the reproducibility of your results. Protocols.io assigns your protocol its own identifier (DOI) so that it can be cited independently in the future. For instructions see: https://journals.plos.org/plosone/s/submission-guidelines#loc-laboratory-protocols. Additionally, PLOS ONE offers an option for publishing peer-reviewed Lab Protocol articles, which describe protocols hosted on protocols.io. Read more information on sharing protocols at https://plos.org/protocols?utm_medium=editorial-email&utm_source=authorletters&utm_campaign=protocols.

We look forward to receiving your revised manuscript.

Kind regards,

Ana Paula Drummond Lage

Academic Editor

PLOS One

Journal Requirements:

When submitting your revision, we need you to address these additional requirements.

1. Please ensure that your manuscript meets PLOS ONE's style requirements, including those for file naming. The PLOS ONE style templates can be found at

https://journals.plos.org/plosone/s/file?id=wjVg/PLOSOne_formatting_sample_main_body.pdf and

https://journals.plos.org/plosone/s/file?id=ba62/PLOSOne_formatting_sample_title_authors_affiliations.pdf

2. PLOS requires an ORCID iD for the corresponding author in Editorial Manager on papers submitted after December 6th, 2016. Please ensure that you have an ORCID iD and that it is validated in Editorial Manager. To do this, go to ‘Update my Information’ (in the upper left-hand corner of the main menu), and click on the Fetch/Validate link next to the ORCID field. This will take you to the ORCID site and allow you to create a new iD or authenticate a pre-existing iD in Editorial Manager.

3. Please include your full ethics statement in the ‘Methods’ section of your manuscript file. In your statement, please include the full name of the IRB or ethics committee who approved or waived your study, as well as whether or not you obtained informed written or verbal consent. If consent was waived for your study, please include this information in your statement as well.

4. Please note that your Data Availability Statement is currently missing a direct link to access each database. If your manuscript is accepted for publication, you will be asked to provide these details on a very short timeline. We therefore suggest that you provide this information now, though we will not hold up the peer review process if you are unable.

5. We note that Figures 4 and 5 in your submission contain [map/satellite] images which may be copyrighted. All PLOS content is published under the Creative Commons Attribution License (CC BY 4.0), which means that the manuscript, images, and Supporting Information files will be freely available online, and any third party is permitted to access, download, copy, distribute, and use these materials in any way, even commercially, with proper attribution. For these reasons, we cannot publish previously copyrighted maps or satellite images created using proprietary data, such as Google software (Google Maps, Street View, and Earth). For more information, see our copyright guidelines: http://journals.plos.org/plosone/s/licenses-and-copyright.

We require you to either (1) present written permission from the copyright holder to publish these figures specifically under the CC BY 4.0 license, or (2) remove the figures from your submission:

(1) You may seek permission from the original copyright holder of Figures 4 and 5 to publish the content specifically under the CC BY 4.0 license.

We recommend that you contact the original copyright holder with the Content Permission Form (http://journals.plos.org/plosone/s/file?id=7c09/content-permission-form.pdf) and the following text:

“I request permission for the open-access journal PLOS ONE to publish XXX under the Creative Commons Attribution License (CCAL) CC BY 4.0 (http://creativecommons.org/licenses/by/4.0/). Please be aware that this license allows unrestricted use and distribution, even commercially, by third parties. Please reply and provide explicit written permission to publish XXX under a CC BY license and complete the attached form.”

Please upload the completed Content Permission Form or other proof of granted permissions as an "Other" file with your submission.

In the figure caption of the copyrighted figure, please include the following text: “Reprinted from [ref] under a CC BY license, with permission from [name of publisher], original copyright [original copyright year].”

(2) If you are unable to obtain permission from the original copyright holder to publish these figures under the CC BY 4.0 license or if the copyright holder’s requirements are incompatible with the CC BY 4.0 license, please either i) remove the figure or ii) supply a replacement figure that complies with the CC BY 4.0 license. Please check copyright information on all replacement figures and update the figure caption with source information. If applicable, please specify in the figure caption text when a figure is similar but not identical to the original image and is therefore for illustrative purposes only.

The following resources for replacing copyrighted map figures may be helpful:

USGS National Map Viewer (public domain): http://viewer.nationalmap.gov/viewer/

The Gateway to Astronaut Photography of Earth (public domain): http://eol.jsc.nasa.gov/sseop/clickmap/

Maps at the CIA (public domain): https://www.cia.gov/library/publications/the-world-factbook/index.html and https://www.cia.gov/library/publications/cia-maps-publications/index.html

NASA Earth Observatory (public domain): http://earthobservatory.nasa.gov/

Landsat: http://landsat.visibleearth.nasa.gov/

USGS EROS (Earth Resources Observatory and Science (EROS) Center) (public domain): http://eros.usgs.gov/#

Natural Earth (public domain): http://www.naturalearthdata.com/

6. If the reviewer comments include a recommendation to cite specific previously published works, please review and evaluate these publications to determine whether they are relevant and should be cited. There is no requirement to cite these works unless the editor has indicated otherwise.

[Note: HTML markup is below. Please do not edit.]

Reviewers' comments:

Reviewer's Responses to Questions

**Comments to the Author**

1. Is the manuscript technically sound, and do the data support the conclusions?

The manuscript must describe a technically sound piece of scientific research with data that supports the conclusions. Experiments must have been conducted rigorously, with appropriate controls, replication, and sample sizes. The conclusions must be drawn appropriately based on the data presented.

Reviewer #1: Partly

Reviewer #2: Yes

**********

2. Has the statistical analysis been performed appropriately and rigorously?

Reviewer #1: No

Reviewer #2: Yes

**********

3. Have the authors made all data underlying the findings in their manuscript fully available?

The PLOS Data policy requires authors to make all data underlying the findings described in their manuscript fully available without restriction, with rare exception (please refer to the Data Availability Statement in the manuscript PDF file). The data should be provided as part of the manuscript or its supporting information, or deposited to a public repository. For example, in addition to summary statistics, the data points behind means, medians and variance measures should be available. If there are restrictions on publicly sharing data—e.g. participant privacy or use of data from a third party—those must be specified.

Reviewer #1: Yes

Reviewer #2: Yes

**********

4. Is the manuscript presented in an intelligible fashion and written in standard English?

PLOS ONE does not copyedit accepted manuscripts, so the language in submitted articles must be clear, correct, and unambiguous. Any typographical or grammatical errors should be corrected at revision, so please note any specific errors here.

Reviewer #1: No

Reviewer #2: Yes

**********

5. Review Comments to the Author

Please use the space provided to explain your answers to the questions above. You may also include additional comments for the author, including concerns about dual publication, research ethics, or publication ethics. (Please upload your review as an attachment if it exceeds 20,000 characters)

Reviewer #1: The manuscript addresses an important topic—spatiotemporal patterns of CRC mortality in China and prediction of future trends—using registry report data (2005–2018) and multiple analytic approaches (Joinpoint regression, age–period–cohort models, Bayesian APC projection, and spatial autocorrelation). The overall direction of the descriptive findings (age gradients, geographic clustering, and differences by sex and urban/rural strata) is plausible.

Major comments

Registry coverage increased markedly over the study period, which may influence the observed temporal trends. Please report the population coverage and number of registries contributing data for each year, and clarify whether the reported rates reflect registry areas only or are intended to represent national estimates. A sensitivity analysis restricted to registries with continuous reporting across the study period would help assess the robustness of the Joinpoint results.

The methods section would benefit from greater detail on how mortality rates and ASMR were calculated. Please specify the age groupings used, the standardization approach (e.g., direct standardization), and whether provincial and urban–rural results are crude or age-standardized. Where feasible, provide uncertainty estimates for annual ASMR values, not only for APC/AAPC.

Additional information is needed on Joinpoint model settings, including the maximum number of joinpoints allowed, the model selection procedure, and the final joinpoint locations for each subgroup. This will improve transparency and reproducibility.

The choice of intrinsic estimator should be briefly justified, with clear description of the age, period, and cohort intervals. The very wide prediction intervals by 2035 suggest substantial model uncertainty; out-of-sample validation or presentation of shorter-term projections as primary results would strengthen confidence in the forecasts. Interpretations of cohort and period effects should also be phrased cautiously given the ecological design.

The manuscript requires substantial English language editing for clarity and grammar, and several sentences over-interpret associations (diet, screening, microbiome) not directly tested in this ecological analysis. Please revise the discussion to frame these as hypotheses consistent with prior literature and expand limitations accordingly.

Reviewer #2: This is a methodologically ambitious, data-rich descriptive epidemiology study suitable for a broad journal audience. Its main strengths lie in comprehensive national surveillance synthesis and pattern visualization. However, the manuscript would benefit from tighter epidemiologic framing, clearer acknowledgment of ecological and surveillance limitations, and more cautious interpretation of APC and predictive findings.

1. Morans I is good but you didnt adjust for confoundsrs. Such as age structure, urbanization, or registry quality.

2. Hotspot regions may reflect better case ascertainment, not higher underlying risk. Spatial findings may conflate surveillance intensity with disease burden.

3. Forecast uncertainty becomes extremely wide after ~2030, with implausibly large confidence intervals (e.g., ASMR CI spanning near zero to >40). Can you do sensitivity analyses (e.g., alternative priors, truncated forecast horizons) as long range predictions are not reliable.

4. Cohort effects peaking in 2010–2014 birth cohorts are difficult to interpret epidemiologically, given limited follow-up time for these cohorts. Period effects (e.g., screening expansion, healthcare reforms) are discussed but not empirically evaluated.

5. Cancer registry coverage expanded substantially over time (~49% population coverage), which may bias temporal trends, especially early increases. Apparent trends may partially reflect surveillance artifacts rather than true mortality changes. Add limitations or correct interpretations

6. My major concenrn is risk of ecological fallacy and over-interpretation of cohort and spatial effects. The study is purely descriptive and ecological, yet portions of the Discussion imply causal interpretations (e.g., diet, screening effects, Westernization). APC and BAPC results should be framed as pattern decomposition and projection, not mechanisms.

**********

6. PLOS authors have the option to publish the peer review history of their article (what does this mean?). If published, this will include your full peer review and any attached files.

If you choose “no”, your identity will remain anonymous but your review may still be made public.

**Do you want your identity to be public for this peer review?**  For information about this choice, including consent withdrawal, please see our Privacy Policy.

Reviewer #1: No

Reviewer #2: No

**********

[NOTE: If reviewer comments were submitted as an attachment file, they will be attached to this email and accessible via the submission site. Please log into your account, locate the manuscript record, and check for the action link "View Attachments". If this link does not appear, there are no attachment files.]

To ensure your figures meet our technical requirements, please review our figure guidelines: https://journals.plos.org/plosone/s/figures

You may also use PLOS’s free figure tool, NAAS, to help you prepare publication quality figures: https://journals.plos.org/plosone/s/figures#loc-tools-for-figure-preparation.

NAAS will assess whether your figures meet our technical requirements by comparing each figure against our figure specifications.

# Author comment: Author response to Decision Letter 1

**DOI:** 10.1371/journal.pone.0352110.r002

12 Apr 2026

PLOS ONE

March 26, 2026

PONE-D-25-66196

"Spatio-temporal patterns and prediction of colorectal cancer mortality in Chinese cancer registration areas: A nationwide study based on multiple models"

Dear Editor and Reviewers:

We are very grateful to receive comments on the revision of the manuscript entitled "Spatio-temporal patterns and prediction of colorectal cancer mortality in Chinese cancer registration areas: A nationwide study based on multiple models" (ID: PONE-D-25-66196).

We sincerely thank all editors and reviewers for their valuable feedback that we have used to clarify the thinking of the study, make the structure of the study more reasonable, and finally improve the quality of our manuscript.

On the separate pages, we provided our response to the comments and suggestions, point by point, and highlighted the changes in the marked copy of the revision. We hope that our revision will be approved by the experts and reviewed favorably.

Sincerely,

Jiageng He

Reviewer Comments:

Reviewer 1

The manuscript addresses an important topic—spatiotemporal patterns of CRC mortality in China and prediction of future trends—using registry report data (2005–2018) and multiple analytic approaches (Joinpoint regression, age–period–cohort models, Bayesian APC projection, and spatial autocorrelation). The overall direction of the descriptive findings (age gradients, geographic clustering, and differences by sex and urban/rural strata) is plausible.

Response: Thank you very much for your recognition and support. We have made revisions to the professional issues, requirements and suggestions you pointed out, and have also improved the entire text. We hope to continue to receive your recognition and professional guidance.

1. Registry coverage increased markedly over the study period, which may influence the observed temporal trends. Please report the population coverage and number of registries contributing data for each year, and clarify whether the reported rates reflect registry areas only or are intended to represent national estimates. A sensitivity analysis restricted to registries with continuous reporting across the study period would help assess the robustness of the Joinpoint results.

Response: We sincerely appreciate these valuable comments. During the course of this study, we supplemented data on the number of registry sites, population size, coverage, and quality control from cancer registry areas in China. The data used in this study were derived from the China Cancer Registry Annual Report. Although the coverage is not 100%, this study includes comprehensive regions across eastern, central, and western China, and currently represents the most reliable data source for reflecting the national cancer epidemiological profile. Since the mortality rates mentioned in the annual report are only those of the cancer registry areas, to ensure greater accuracy and a more cautious expression, the authors have restricted the geographic description throughout the manuscript to “Chinese cancer registry areas.” Of course, this is also one of the limitations of this study. We have mentioned the limitation in the section on limitations. We sincerely hope for your understanding and approval.

The revised content has been highlighted in blue.

In lines 85-110:

Data quality

To ensure the authenticity, stability, and comparability of the data, data collection, verification, and analysis were conducted in accordance with the Chinese Cancer Registry Guidelines and the standards of the IARC, ensuring consistency in registration procedures and coding practices across regions and study years. All registries underwent standardized evaluations of comparability, completeness, and validity.

The inclusion and exclusion of data strictly adhered to the quality control standards for Chinese cancer registration work. Data included in the report must meet the following core quality requirements: the morphological verification percentage (MV%) should range between 55% and 95%; the percentage of death certificate only (DCO%) should be less than 20%; the mortality-to-incidence ratio (M/I) should fall within the range of 0.55 to 0.85.

From 2008 to 2021, the coverage of cancer registries reported in the China Cancer Registry Annual Report expanded steadily. It increased from 45 cancer registries in 2005, covering a population of 69,369,668 (accounting for 5.31% of the national population at the end of 2005), to 947 registries by 2018, covering 634,376,540 individuals (representing 45.14% of the national population at the end of 2018) (Table 1). From 2005 to 2018, the MV%, DCO%, and the M/I were 67.83%, 2.11%, and 0.62, respectively, all of which met national quality benchmarks (Table 2). These indicators collectively demonstrate that the registry data are robust and reliable, providing a sound foundation for epidemiological analysis.

Table 1 Number of cancer registries and population coverage in China, 2005–2018

Year Number of cancer registries Covered population Population coverage (%)

2005 45 69,369,668 5.31

2006 49 76,209,748 5.80

2007 48 70,782,375 5.36

2008 56 82,433,497 6.21

2009 104 109,476,347 8.20

2010 219 207,229,403 15.42

2011 234 221,390,275 16.43

2012 261 239,887,749 17.64

2013 347 287,284,044 21.11

2014 449 345,711,646 25.27

2015 501 387,872,825 28.22

2016 682 476,692,113 34.47

2017 821 563,934,185 40.57

2018 947 634,376,540 45.14

Table 2 Quality indicators of cancer registry data in China, 2005–2018

Year National Urban Rural

MV(%) DCO(%) M/I MV(%) DCO(%) M/I MV(%) DCO(%) M/I

2005 66.00 1.76 0.65 69.02 2.08 0.65 56.09 0.71 0.28

2006 65.59 1.66 0.64 67.71 1.85 0.61 57.11 0.90 0.76

2007 65.83 1.95 0.64 67.71 2.28 0.61 59.57 0.83 0.75

2008 69.33 2.23 0.62 70.53 2.49 0.59 64.22 1.12 0.73

2009 67.23 3.14 0.63 68.96 3.03 0.60 62.91 3.43 0.71

2010 67.11 2.99 0.61 71.51 2.49 0.59 60.65 3.72 0.64

2011 70.14 2.44 0.63 72.92 2.17 0.61 65.34 2.90 0.67

2012 69.13 2.38 0.62 70.63 2.63 0.59 67.31 2.09 0.65

2013 68.04 1.74 0.62 70.76 1.76 0.59 64.97 1.72 0.65

2014 68.01 2.91 0.61 69.75 2.79 0.58 65.96 1.49 0.64

2015 39.31 2.08 0.61 64.45 3.25 0.53 62.32 2.92 0.63

2016 68.31 1.40 0.61 70.03 1.58 0.59 66.29 1.18 0.63

2017 67.77 1.53 0.59 69.97 1.62 0.56 65.47 1.44 0.62

2018 69.94 1.34 0.58 71.50 1.58 0.55 68.48 1.11 0.61

AVG 65.84 2.11 0.62 69.68 2.26 0.59 63.34 1.83 0.64

MV%, morphological verification percentage; DCO%, percentage of death certificate only; M/I, mortality-to-incidence ratio.

2. The methods section would benefit from greater detail on how mortality rates and ASMR were calculated. Please specify the age groupings used, the standardization approach (e.g., direct standardization), and whether provincial and urban–rural results are crude or age-standardized. Where feasible, provide uncertainty estimates for annual ASMR values, not only for APC/AAPC.

Response: We sincerely appreciate the valuable comments. We have added the calculation formula for ASMR in the method. When analyzing 31 provincial-level administrative regions, only crude mortality rates were used due to data limitations. All other methods used age-standardized mortality rates for calculation, and we also supplemented the 95% confidence interval of ASMR as suggested.

The revised content has been highlighted in blue.

In lines 116-140:

Statistical indicators

Mortality rate, also known as crude mortality rate, refers to the number of cancer deaths registered per 100,000 population in a given year, and reflects the mortality level of the population. Since the crude mortality rate is significantly affected by the age composition of the population, when comparing and analyzing mortality rates across different regions or the mortality levels of the same population group over different periods, it is necessary to calculate the age-standardized mortality rate (ASMR) to eliminate the impact of population age structure on mortality. The ASMR refers to the mortality rate calculated according to the age structure of a standard population. In this study, mortality data across the country, different genders, and different regions were divided into 18 age groups with an interval of 5 years: 0-4 years, 5-9 years, 10-14 years...80-84 years, and 85 years and above. The standard population data came from the sixth national population census data released by the National Bureau of Statistics of China in 2010[21]. In this study, ASMR were used in the Joinpoint regression and BAPC models to ensure comparability over time and across populations. For provincial-level spatial analysis, crude mortality rates were used due to the lack of age-specific data in China Cancer Registry Annual Reports.

Mortality rate per 100,000=countpopulation×100,000

ASMR=standard population in corresponding age group × age-specific mortality ratestandard population×100,000

The 95% confidence interval (CI) for the ASMR was estimated using the Fay-Feuer method, which is robust for directly standardized rates across varying population sizes and incidence levels[23].

ASMR 95%CIlow=v2×ASMR×ChiInv0.052,(2×ASMR2)v×100,000

ASMR 95%CIhigh=v+wm22×(ASMR+wm)×ChiInv1-0.052,2(ASMR+wm)2v+wm2×100,000

Where, wm is the weight correction factor, v is the estimated variance of the ASMR, ChiInv (p, n) is the inverse of the chi-squared distribution function evaluated at p and with n degrees of freedom.

Table 3 Mortality rate of CRC in Chinese cancer registration areas from 2005 to 2018 (/100,000)

Years National Male Female Urban Rural

Deaths Rates ASMR

(95%CI) Deaths Rates ASMR

(95%CI) Deaths Rates ASMR

(95%CI) Deaths Rates ASMR

(95%CI) Deaths Rates ASMR

(95%CI)

2005 7,049 12.83 11.40

(11.08, 11.62) 3,877 13.93 13.49

(13.00, 13.86) 3,172 11.71 9.60

(9.22, 9.90) 5,901 14.51 12.23

(12.10, 12.45) 1,149 8.07 8.45

(8.35, 8.56)

2006 7,979 13.40 11.78

(11.47, 11.99) 4,349 14.49 13.87

(13.38, 14.21) 3,632 12.29 10.06

(9.68, 10.34) 6,934 14.89 12.51

(12.38, 12.61) 1,044 8.03 8.46

(8.30, 8.57)

2007 8,475 14.17 12.19

(11.88, 12.40) 4,711 15.58 14.50

(14.02, 14.85) 3,754 12.69 10.14

(9.76, 10.42) 7,128 15.98 12.97

(12.87, 13.10) 1,344 8.84 9.19

(9.09, 9.28)

2008 9,800 14.82 11.84

(11.55, 12.02) 5,213 15.64 13.52

(13.09, 13.82) 4,585 13.98 10.39

(10.04, 10.65) 8,575 16.44 12.64

(12.45, 12.80) 1,224 8.75 8.17

(8.05, 8.30)

2009 12,163 14.23 11.83

(11.56, 11.98) 6,801 15.73 14.05

(13.64, 14.31) 5,361 12.69 9.85

(9.53, 10.06) 9,819 17.08 13.51

(13.41, 13.70) 2,527 9.03 8.62

(8.54, 8.71)

2010 16,679 23.52 11.30

(11.07, 11.42) 9,492 15.05 13.70

(13.36, 13.91) 7,186 11.67 9.19

(9.09, 10.49) 12,757 15.95 12.85

(12.65, 12.98) 3,921 8.78 8.09

(8.00, 8.20)

2011 19,746 13.55 11.31

(11.10, 11.41) 11,297 15.34 13.74

(13.42, 13.93) 8,446 11.71 9.13

(8.88, 9.28) 14,404 16.46 13.01

(12.88, 13.11) 5,172 9.66 8.98

(8.90, 9.07)

2012 25,553 12.90 10.68

(10.50, 10.76) 14,546 14.49 12.86

(12.59, 13.00) 11,003 11.26 8.70

(8.49, 8.82) 16,247 16.17 12.29

(12.15, 12.41) 9,300 9.53 8.63

(8.51, 8.71)

2013 29,502 13.03 10.52

(10.34, 10.59) 16,794 14.62 12.63

(12.37, 12.75) 12,712 11.39 8.60

(8.41, 8.71) 18,363 16.45 12.22

(12.02, 12.44) 11,142 9.70 8.53

(8.45, 8.60)

2014 38,268 13.28 10.50

(10.35, 10.56) 22,116 15.13 12.72

(12.49, 12.83) 16,150 11.37 8.46

(8.29, 8.55) 23,745 16.48 12.12

(12.00, 12.41) 14,518 10.07 8.61

(8.55, 8.70)

2015 44,362 13.82 10.60

(10.45, 10.65) 25,950 15.94 12.99

(12.77, 13.09) 18,427 11.65 8.39

(8.09, 8.97) 26,360 17.10 12.26

(12.06, 12.50) 18,004 10.79 8.83

(8.73, 8.91)

2016 53,811 14.10 10.64

(10.50, 10.69) 31,552 16.30 13.05

(12.84, 13.13) 22,243 11.84 8.40

(8.25, 8.47) 32,750 17.00 12.16

(11.99, 12.31) 21,072 11.15 8.91

(8.85, 8.99)

2017 61,451 14.08 10.53

(10.39, 10.56) 36,203 16.37 12.99

(12.79, 13.06) 25,235 11.73 8.25

(8.10, 8.31) 35,838 16.81 11.93

(11.87, 12.06) 25,598 11.47 9.03

(8.96, 9.10)

2018 75,969 14.52 10.65

(10.52, 10.68) 44,989 16.95 13.25

(13.05, 13.3) 30,975 12.02 8.26

(8.12, 8.31) 40,192 17.03 11.89

(11.75, 12.06) 35,767 12.46 9.54

(9.44, 9.60)

Total 410,807 14.45 11.13

(11.03, 11.27) 237,890 15.40 13.38

(13.05, 13.61) 172,881 12.00 9.10

(8.97, 9.15) 259,013 16.31 12.47

(12.27, 12.60) 151,782 9.74 8.72

(8.65, 8.78)

CRC, colorectal cancer; ASMR, age-standardized mortality rate.

3. Additional information is needed on Joinpoint model settings, including the maximum number of joinpoints allowed, the model selection procedure, and the final joinpoint locations for each subgroup. This will improve transparency and reproducibility.

Response: We sincerely appreciate the valuable comments. In this study, Joinpoint regression analysis was conducted using the Joinpoint regression program. The maximum number of joinpoints is set to 4. Model selection is performed using Monte Carlo permutation test, which is the default and recommended method in Joinpoint software. The final number and location of connection points for each subgroup are automatically determined by the model selection process described above. We have supplemented it in the methods, The revised content has been highlighted in blue.

In lines 142-166:

Joinpoint regression model

Joinpoint regression was applied to analyze temporal trends in CRC mortality. This method fits segmented regression models to time-series data and identifies points where statistically significant changes in trends occur. Given that cancer mortality data typically follow a Poisson or exponential distribution, a log-linear Joinpoint model was employed in this study[24, 25]. The model can be expressed as:

lnRy=β0+β1y+k=1Kδky−τk+

Where, Ry denotes the CRC mortality (per 100,000 population) in year y, β0 is the intercept, β1 is the baseline slope, τk represents the location of the k-th joinpoint, and (y−τk)+=max(0, y−τk). The number of joinpoints (K) was set to four in this study.

Model selection was performed using the Monte Carlo permutation test, which identifies the optimal number of joinpoints by comparing goodness-of-fit across models.

The period 2005–2018 was divided into multiple segments. The annual percentage change (APC) was used to evaluate within-segment trends, while the average annual percentage change (AAPC) described the overall trend. A 95% CI was used to assess statistical significance. When the number of joinpoints equals zero, APC=AAPC. APC>0 or AAPC>0 indicates an increasing trend, while APC<0 or AAPC<0 indicates a decreasing trend.

APC calculation formula:

APC=eβ1−1×100

AAPC calculation formula:

AAPC=expωiβi/ωi−1×100

In the formula, β1 is the regression coefficient, βi is the regression coefficient corresponding to each interval and ωi is the span of each interval (i.e. the number of years included in the interval).

4. The choice of intrinsic estimator should be briefly justified, with clear description of the age, period, and cohort intervals. The very wide prediction intervals by 2035 suggest substantial model uncertainty; out-of-sample validation or presentation of shorter-term projections as primary results would strengthen confidence in the forecasts. Interpretations of cohort and period effects should also be phrased cautiously given the ecological design.

Response: We sincerely appreciate the valuable comments. We provided a more detailed explanation of the age-period-cohort and BAPC model, and to ensure the accuracy and reliability of the results, we conducted sensitivity analysis using GBD data from the same period. The results also yielded similar conclusions to our structure.

The revised content has been highlighted in blue.

In lines 167-191:

Age–period–cohort model

The age–period–cohort model is based on the Poisson distribution and is used to identify the independent effects of age, period, and cohort on observed variables[26]. The model is expressed as:

logλapc=αa+βp+γc+ε

In the formula, α、β and γ represent the effects of age, period, and cohort, while ε represents the residual.

According to the requirements of the age-period-cohort model, the data needs to be organized into the following format:

Age group

This study included data on CRC deaths in various age groups from 2005 to 2018. The CRC death cases in China were divid

Attachment Submitted filename: Response to Reviewers.docx

# Peer review: Decision Letter 1

**DOI:** 10.1371/journal.pone.0352110.r003

**Authors:** Ana Paula Drummond Lage, Ana Paula Drummond Lage

4 Jun 2026

Spatio-temporal patterns and prediction of colorectal cancer mortality in Chinese cancer registration areas: A nationwide study based on multiple models

PONE-D-25-66196R1

Dear Dr.He,

We’re pleased to inform you that your manuscript has been judged scientifically suitable for publication and will be formally accepted for publication once it meets all outstanding technical requirements.

Within one week, you’ll receive an e-mail detailing the required amendments. When these have been addressed, you’ll receive a formal acceptance letter and your manuscript will be scheduled for publication.

An invoice will be generated when your article is formally accepted. Please note, if your institution has a publishing partnership with PLOS and your article meets the relevant criteria, all or part of your publication costs will be covered. Please make sure your user information is up-to-date by logging into Editorial Manager at Editorial Manager® and clicking the ‘Update My Information' link at the top of the page. For questions related to billing, please contact billing support.

If your institution or institutions have a press office, please notify them about your upcoming paper to help maximize its impact. If they’ll be preparing press materials, please inform our press team as soon as possible -- no later than 48 hours after receiving the formal acceptance. Your manuscript will remain under strict press embargo until 2 pm Eastern Time on the date of publication. For more information, please contact onepress@plos.org.

Kind regards,

Ana Paula Drummond Lage

Academic Editor

PLOS One

Additional Editor Comments (optional):

Thank you for submitting the revised version of your manuscript. We have carefully considered the revisions and the responses provided to the reviewers' comments.

We are pleased to note that the authors have addressed all of the reviewers' concerns in a thoughtful and satisfactory manner. The revisions have strengthened the manuscript and improved its clarity and overall quality.

In light of these changes, and considering that all relevant issues raised during the peer-review process have been adequately resolved, we are pleased to accept the manuscript for publication.

We appreciate the authors' careful attention to the reviewers' suggestions and thank them for their contribution.

Reviewers' comments:

Reviewer's Responses to Questions

**Comments to the Author**

1. If the authors have adequately addressed your comments raised in a previous round of review and you feel that this manuscript is now acceptable for publication, you may indicate that here to bypass the “Comments to the Author” section, enter your conflict of interest statement in the “Confidential to Editor” section, and submit your "Accept" recommendation.

Reviewer #2: All comments have been addressed

**********

2. Is the manuscript technically sound, and do the data support the conclusions?

The manuscript must describe a technically sound piece of scientific research with data that supports the conclusions. Experiments must have been conducted rigorously, with appropriate controls, replication, and sample sizes. The conclusions must be drawn appropriately based on the data presented.

Reviewer #2: Yes

**********

3. Has the statistical analysis been performed appropriately and rigorously?

Reviewer #2: Yes

**********

4. Have the authors made all data underlying the findings in their manuscript fully available?

The PLOS Data policy requires authors to make all data underlying the findings described in their manuscript fully available without restriction, with rare exception (please refer to the Data Availability Statement in the manuscript PDF file). The data should be provided as part of the manuscript or its supporting information, or deposited to a public repository. For example, in addition to summary statistics, the data points behind means, medians and variance measures should be available. If there are restrictions on publicly sharing data—e.g. participant privacy or use of data from a third party—those must be specified.

Reviewer #2: Yes

**********

5. Is the manuscript presented in an intelligible fashion and written in standard English?

PLOS ONE does not copyedit accepted manuscripts, so the language in submitted articles must be clear, correct, and unambiguous. Any typographical or grammatical errors should be corrected at revision, so please note any specific errors here.

Reviewer #2: Yes

**********

6. Review Comments to the Author

Please use the space provided to explain your answers to the questions above. You may also include additional comments for the author, including concerns about dual publication, research ethics, or publication ethics. (Please upload your review as an attachment if it exceeds 20,000 characters)

Reviewer #2: Thank you for addressing my comments; the study now looks much clearer and stronger overall. Appreciate the revisions done.

**********

7. PLOS authors have the option to publish the peer review history of their article (what does this mean?). If published, this will include your full peer review and any attached files.

If you choose “no”, your identity will remain anonymous but your review may still be made public.

**Do you want your identity to be public for this peer review?**  For information about this choice, including consent withdrawal, please see our Privacy Policy.

Reviewer #2: No

**********

# Editor report: Acceptance letter

**DOI:** 10.1371/journal.pone.0352110.r004

**Authors:** Ana Paula Drummond Lage, Ana Paula Drummond Lage

PONE-D-25-66196R1

PLOS One

Dear Dr. He,

I'm pleased to inform you that your manuscript has been deemed suitable for publication in PLOS One. Congratulations! Your manuscript is now being handed over to our production team.

At this stage, our production department will prepare your paper for publication. This includes ensuring the following:

* All references, tables, and figures are properly cited

* All relevant supporting information is included in the manuscript submission,

* There are no issues that prevent the paper from being properly typeset

You will receive further instructions from the production team, including instructions on how to review your proof when it is ready. Please keep in mind that we are working through a large volume of accepted articles, so please give us a few days to review your paper and let you know the next and final steps.

Lastly, if your institution or institutions have a press office, please let them know about your upcoming paper now to help maximize its impact. If they'll be preparing press materials, please inform our press team within the next 48 hours. Your manuscript will remain under strict press embargo until 2 pm Eastern Time on the date of publication. For more information, please contact onepress@plos.org.

You will receive an invoice from PLOS for your publication fee after your manuscript has reached the completed accept phase. If you receive an email requesting payment before acceptance or for any other service, this may be a phishing scheme. Learn how to identify phishing emails and protect your accounts at https://explore.plos.org/phishing.

If we can help with anything else, please email us at customercare@plos.org.

Thank you for submitting your work to PLOS ONE and supporting open access.

Kind regards,

PLOS ONE Editorial Office Staff

on behalf of

Dr. Ana Paula Drummond Lage

Academic Editor

PLOS One
